# Assessing SOFA score trajectories in sepsis using machine learning: A pragmatic approach to improve the accuracy of mortality prediction

**DOI:** 10.1371/journal.pone.0300739

**Published:** 2024-03-28

**Authors:** Lars Palmowski, Hartmuth Nowak, Andrea Witowski, Björn Koos, Alexander Wolf, Maike Weber, Daniel Kleefisch, Matthias Unterberg, Helge Haberl, Alexander von Busch, Christian Ertmer, Alexander Zarbock, Christian Bode, Christian Putensen, Ulrich Limper, Frank Wappler, Thomas Köhler, Dietrich Henzler, Daniel Oswald, Björn Ellger, Stefan F. Ehrentraut, Lars Bergmann, Katharina Rump, Dominik Ziehe, Nina Babel, Barbara Sitek, Katrin Marcus, Ulrich H. Frey, Patrick J. Thoral, Michael Adamzik, Martin Eisenacher, Tim Rahmel, on behalf of the SepsisDataNet.NRW research group

**Affiliations:** 1 Klinik für Anästhesiologie, Intensivmedizin und Schmerztherapie, Universitätsklinikum Knappschaftskrankenhaus Bochum, Ruhr Universität Bochum, Bochum, Germany; 2 Zentrum für Künstliche Intelligenz, Medizininformatik und Datenwissenschaften, Universitätsklinikum Knappschaftskrankenhaus Bochum, Bochum, Germany; 3 Medizinische Fakultät, Medizinisches Proteom-Center, Ruhr Universität Bochum, Bochum, Germany; 4 Zentrum für Proteindiagnostik (PRODI), Ruhr Universität Bochum, Bochum, Germany; 5 Klinik für Anästhesiologie, Operative Intensivmedizin und Schmerztherapie, Universitätsklinikum Münster, Münster, Germany; 6 Klinik für Anästhesiologie und Operative Intensivmedizin, Universitätsklinikum Bonn, Bonn, Germany; 7 Klinik für Anästhesiologie und Operative Intensivmedizin, Universität Witten/Herdecke, Krankenhaus Köln-Merheim, Köln, Germany; 8 Klinik für Anästhesiologie und Operative Intensiv-, Rettungsmedizin und Schmerztherapie, Klinikum Herford, Herford, Germany; 9 Klinik für Anästhesiologie und Intensivmedizin, AMEOS-Klinikum Halberstadt, Halberstadt, Germany; 10 Klinik für Anästhesiologie, Intensivmedizin und Schmerztherapie, Klinikum Westfalen, Dortmund, Germany; 11 Centrum für Translationale Medizin, Medizinische Klinik I, Marien Hospital Herne, Universitätsklinikum der Ruhr-Universität Bochum, Herne, Germany; 12 Klinik für Anästhesiologie, Operative Intensivmedizin, Schmerz- und Palliativmedizin, Marien Hospital Herne, Universitätsklinikum der Ruhr-Universität Bochum, Bochum, Germany; 13 Department of Intensive Care Medicine, Laboratory for Critical Care Computational Intelligence, Amsterdam Cardiovascular Science (ACS), Amsterdam Infection and Immunity Institute (AI&II), Amsterdam UMC, Location VUmc, Vrije Universiteit Amsterdam, Amsterdam, The Netherlands; Azienda Ospedaliero Universitaria Careggi, ITALY

## Abstract

**Introduction:**

An increasing amount of longitudinal health data is available on critically ill septic patients in the age of digital medicine, including daily sequential organ failure assessment (SOFA) score measurements. Thus, the assessment in sepsis focuses increasingly on the evaluation of the individual disease’s trajectory. Machine learning (ML) algorithms may provide a promising approach here to improve the evaluation of daily SOFA score dynamics. We tested whether ML algorithms can outperform the conventional ΔSOFA score regarding the accuracy of 30-day mortality prediction.

**Methods:**

We used the multicentric SepsisDataNet.NRW study cohort that prospectively enrolled 252 sepsis patients between 03/2018 and 09/2019 for training ML algorithms, i.e. support vector machine (SVM) with polynomial kernel and artificial neural network (aNN). We used the Amsterdam UMC database covering 1,790 sepsis patients for external and independent validation.

**Results:**

Both SVM (AUC 0.84; 95% CI: 0.71–0.96) and aNN (AUC 0.82; 95% CI: 0.69–0.95) assessing the SOFA scores of the first seven days led to a more accurate prognosis of 30-day mortality compared to the ΔSOFA score between day 1 and 7 (AUC 0.73; 95% CI: 0.65–0.80; p = 0.02 and p = 0.05, respectively). These differences were even more prominent the shorter the time interval considered. Using the SOFA scores of day 1 to 3 SVM (AUC 0.82; 95% CI: 0.68 0.95) and aNN (AUC 0.80; 95% CI: 0.660.93) led to a more accurate prognosis of 30-day mortality compared to the ΔSOFA score (AUC 0.66; 95% CI: 0.58–0.74; p < 0.01 and p < 0.01, respectively). Strikingly, all these findings could be confirmed in the independent external validation cohort.

**Conclusions:**

The ML-based algorithms using daily SOFA scores markedly improved the accuracy of mortality compared to the conventional ΔSOFA score. Therefore, this approach could provide a promising and automated approach to assess the individual disease trajectory in sepsis. These findings reflect the potential of incorporating ML algorithms as robust and generalizable support tools on intensive care units.

## Introduction

The sequential organ failure assessment (SOFA) score has been widely used in the evaluation of critical ill patients since its development in the early 1990s. As one of the most commonly used scoring systems in clinical practice on intensive care units (ICUs), the SOFA score has been demonstrated as an effective tool for evaluating the prognosis of patients suffering from sepsis [1, 2]. With the Sepsis-3 definition, the SOFA score became a key element in identifying sepsis and is now a major criterion in diagnosing sepsis [3]. Furthermore, it objectively describes the degree of a sepsis-associated organ dysfunction, helping to evaluate disease severity and progression [4].

The daily assessment of patients’ organ dysfunction by using the SOFA score in the ICU represents a valuable outcome indicator [4]. In addition, it is increasingly used to determine the efficacy of novel therapeutic agents, and is accepted by the European Medicines Agency as an endpoint in exploratory trials [5]. Daily SOFA scores are more and more routinely documented as longitudinal health records in sepsis patients, offering robust information about the disease trajectory and therapeutic success. However, the evaluation of longitudinal SOFA score data should be performed in a standardized manner supporting generalizability.

The trajectory of the SOFA score can be described by several approaches of which the ΔSOFA is the most popular [2]. The change between the SOFA score at ICU admission and a day in the further course is most frequently used. In this context, the ΔSOFA score between day 1 and 7 was able to predict the 28-day prognosis of sepsis patients [6]. However, using the ΔSOFA also has potential limitations as it does not sufficiently reflect the baseline severity and neglects the alterations of the days between the delta, which may limit the generalizability of ΔSOFA prediction models [7, 8].

Machine learning (ML) has also been utilized with increasing frequency in the field of medicine in recent years, and is regarded as a robust and effective method, especially in reflecting individual disease trajectories [9–12]. Several studies have convincingly demonstrated that ML models can outperform traditional clinical scoring approaches, including the prediction of mortality risk in septic patients [13, 14]. Thus, implementing ML algorithms in assessing the longitudinal measurement of the SOFA score may help to overcome several limitations inherent in the ΔSOFA approach. The assessment of longitudinal data, including the daily SOFA score, by using ML algorithms no longer represents a technical challenge in the digital age of medicine, with the widespread availability of electronic health records. Thus, ML-based models to evaluate longitudinal SOFA score data could offer a promising opportunity to apply computational approaches beyond existing conventional ΔSOFA models that are translatable into clinical routine.

Therefore, we tested the hypothesis that ML-based analysis of daily SOFA scores covering the first seven days after sepsis diagnosis leads to a more accurate prediction of 30-day mortality than using the conventional ΔSOFA approach.

## Materials and methods

### Study design and conceptual overview

In this study, we analyzed two independent non-overlapping intensive care cohorts. The first was the prospectively enrolled, multicentric study cohort that was used to train (derivation cohort) the ML algorithms to predict 30-day mortality using daily SOFA scores between day 1 and 7. The second was the retrospective monocentric database that was used as a validation cohort. The ML-based algorithms were each compared to the ΔSOFA score of day 1 to 7, as described by Karakike [6]. In addition, shortened time intervals were additionally examined between day 1 and 3 as well as day 1 and 5, and compared with the respective ΔSOFA score.

### Study cohorts

**Fig 1:**
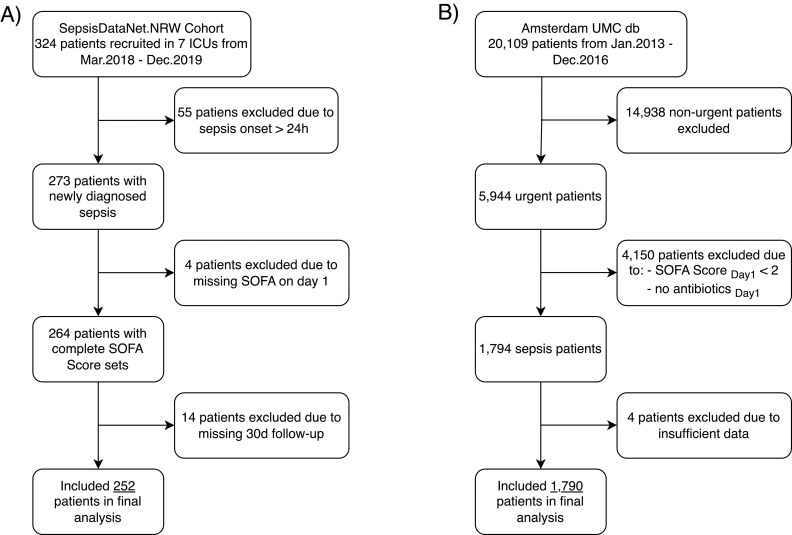
Flow charts for the identification of the final analysis cohort. a) 54 patients were excluded from 324 patients recruited to the SepsisDataNet.NRW study due to onset of sepsis > 24h ago after recruitment, while another 4 patients were excluded due missing SOFA Score on day 1, which was essential for our approach. Finally, 14 patients were excluded due to missing 30d follow-up. The remaining 252 patients were included in the analysis. b) Of the 20,109 unique patients in the Amsterdam UMCdb 14,165 were excluded because they were classified as ‘non-urgent.’ Furthermore, 4,150 patients were excluded because they did not meet the sepsis 3 criteria and another 4 patients were excluded due to missing data. The resulting 1,790 patients were included in the analysis.

The first cohort consisted of prospectively enrolled critically ill adult septic patients fulfilling the Sepsis-3 criteria as part of the multicentric SepsisDataNet.NRW study (German clinical trial registry: DRKS00018871, http://www.sepsisdatanet.nrw). These patients were recruited over a two-year period between March 1, 2018, and December 31, 2019 in the ICUs of seven German university hospitals. After ethical approval (Ruhr-University Bochum, registry number 5047–14), 252 patients were included in this study following the acquisition of written informed consent. Subsequently, these patients were considered for the final analysis using the clinical parameters listed in the ethical approval and study protocol. The study protocol of the SepsisDataNet.NRW cohort allowed patients to be included up to 72 h after sepsis diagnosis. Since the SOFA score from day 1 is of major importance in our work, we only considered patients in our analysis, who were included into the prospective cohort on day one of sepsis diagnosis (Fig 1).

Study data were used to calculate the daily SOFA score and assessment of 30-day mortality. The SOFA score was calculated by a local independent physician at each site and validated against an automatically derived SOFA score based on the documentation in the study database. In case of non-matching scores, the SOFA score was additionally determined by a second independent physician, in order to clarify the inconsistency. All methods were performed following the relevant guidelines and regulations. All research has been performed in accordance with the Declaration of Helsinki.

The validation cohort consisted of a large retrospective database of intensive care admissions from the Amsterdam UMC between January 1, 2003, and December 31, 2016 (last access date: September 14, 2020). This is a publicly accessible database that, following privacy and ethics audits, provides anonymized patient data for research purposes [15]. Septic patients were identified in this database as described by Komorowski et al. [16]. Only ‘urgent’ patients in need of antibiotic treatment and a SOFA score of 2 or more were considered as septic patients. ‘Non-urgent’ patients, patients without antibiotic treatment or with SOFA score < 2 on day 1 were excluded. Age < 18 years at the time of ICU admission, non-documented 30-day survival status, insufficient data collection for the calculation of the SOFA score or readmission to the ICU within the study period led to exclusion in both cohorts (Fig 1). The number of missing values was <1% in the derivation cohort and about 8% in the validation cohort. For missing values (except for SOFA score day 1, which led to exclusion if missing), the last available SOFA score was carried forward to handle missing values. The patients in both cohorts were anonymized. The authors had no access to information that could identify individual participants.

### Data extraction and preprocessing

Hospital information system data were extracted for each patient, including the parameters for calculating the SOFA score. If multiple SOFA score assessments were available per 24-h period, according to our predefined data processing approach, the worst value (highest SOFA score value) per day was used for the study analysis.

### Calculation of the SOFA score

The components of the SOFA score (central nervous system, renal, liver, cardiovascular, respiratory and coagulation) for each 24 h period were calculated for the first seven days. We strictly followed the general guidelines and proposals for calculating the SOFA score as described by Lambden et al. [17]. Regarding sedated patients, the last pre-intubation Glasgow Coma Score was carried forward throughout sedation. If the Glasgow Coma Score prior to sedation was unknown, a value of 15/15 was recorded and carried forward to all following days ensuing intubation. The last available SOFA score was carried forward until day 7 in case of death or ICU discharge before day 7. We calculated the ΔSOFA score using the previously described procedure as ΔSOFA = (SOFA day x—SOFA day 1) * 100 / SOFA day 1, where day x was either day 3, 5, or 7 [6].

### Development of the classification model

The SOFA scores for each of the first seven days after the diagnosis of sepsis were used as features to develop and compare different classification models based on the prospective derivation cohort. All models aimed to predict the 30-day survival of the sepsis patients included. Although additional variables would have likely improved mortality prediction accuracy, all ML-based models were limited to the use of the summarized value of the SOFA score (i.e the total sum) as an input variable to allow a direct comparison with the ΔSOFA approach and maintaining simplicity.

Various ML algorithms were compared to optimize the prediction model. We used logistic regression, linear discriminant analysis, random forest, support vector machines (SVM) with linear kernel, SVM with polynomial kernel, artificial neural networks (aNN) and aNN with feature extraction using a principal component analysis.

**Fig 2:**
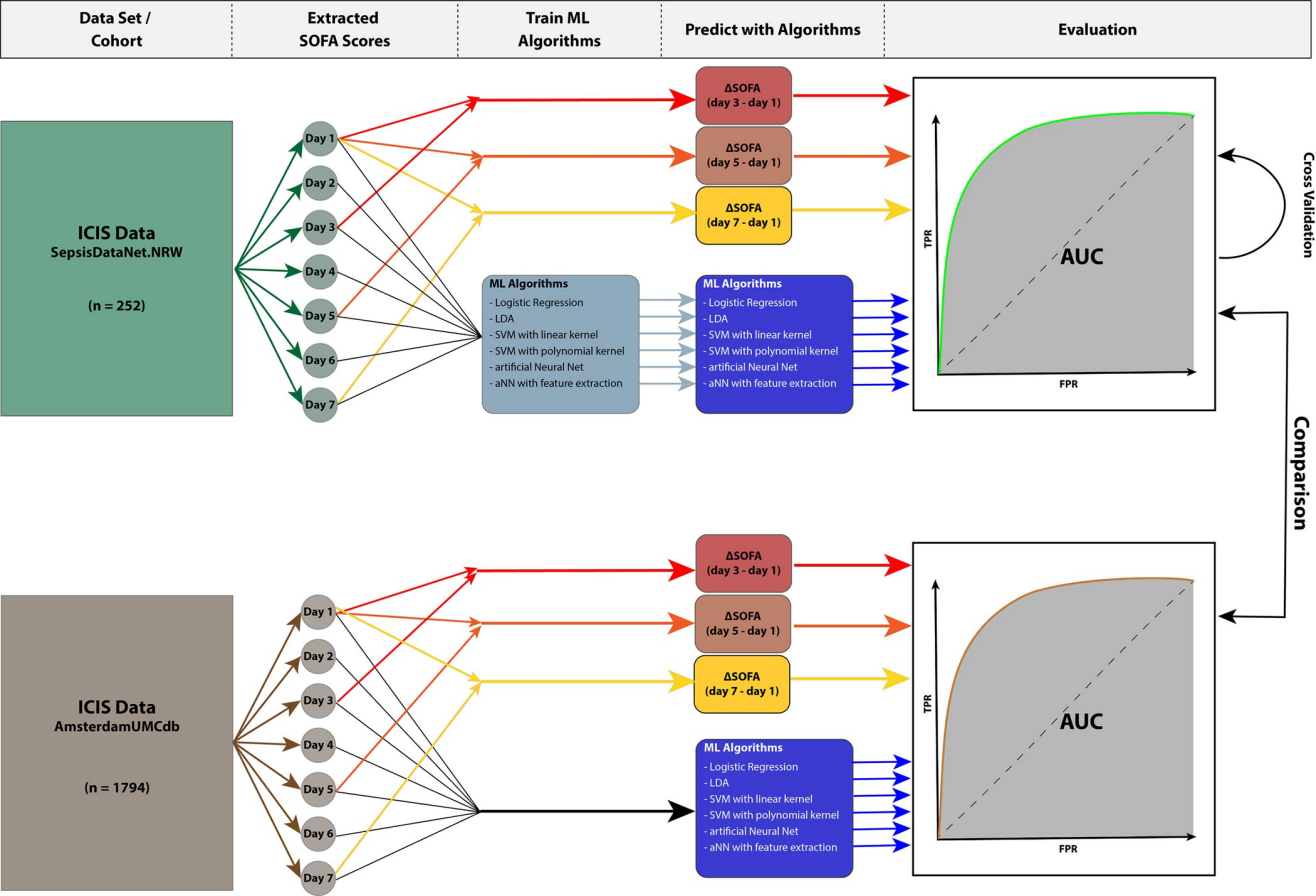
Flow of the data extracted from the ICIS databases. Firstly, the raw data was extracted and the SOFA scores per day for the first seven days were calculated. These scores were used as features in the machine learning (ML) process. The ML algorithms were trained and then tested. The test results, in the form of receiver operator characteristics (ROCs) were evaluated and statistically compared to the ΔSOFA score. The same workflow was done for the data extracted from the validation cohort with the exception of the ML training process. The ROCs yielded from this validation cohort were compared to those from the derivation cohort.

The models were trained with a five times repeated 10-fold cross-validation approach to reduce bias and prevent overfitting [18]. The distributions of the AUC within the cross-validation are plotted as histograms for all models in S1 File. The area under the receiver operator characteristic curve (AUROC) was used as the optimization criterion for the optimization of hyperparameters. The mean AUROCs after cross validation and its 95% confidence intervals (CIs) were used to compare the different classification models. Each model was validated using the retrospective validation cohort (Fig 2). In addition, each of the classification algorithms was trained using only the SOFA scores of days 1 to 5 as features (classification model with five features) as well as the SOFA scores of days 1 to 3 (classification model with three features). The procedure for these models with smaller feature subsets was the same as for those with the SOFA scores of the first seven days. All developed classifier models and instructions on how to apply them can be accessed in S2 File.

### Statistics

Continuous variables are presented as means ± standard deviation in the case of normal distribution and as median and interquartile range (25th; 75th percentile) in the case of non-normally distributed variables. The AUROCs of the different classification models were compared using the method described by Hanley et al. [19]. This method was chosen for its suitability in evaluating paired outcomes, a relevant consideration in our study where models were applied to the same dataset. Hanley’s approach accounts for the pairing of results, providing a robust assessment of differences in discriminatory performance among various models. In a second step, a receiver operator characteristic (ROC) analysis of 30-day mortality was used to define ΔSOFA cutoff values with the Youden Index to discriminate between predicted survivors and predicted deaths for each time period assessed. Regarding the ML models, the cutoff was set at 0.5. If the predicted probability of mortality within 30 days was greater than 0.5, the patient was classified as deceased. Survival analysis were graphically assessed by Kaplan-Meier curves. The sensitivity, specificity, and the positive and negative predictive value were calculated for each algorithm. Reclassification analyses using net reclassification improvement (NRI) and integrated discrimination improvement (IDI) were used to assess the added value of ML-based algorithms compared to the corresponding ΔSOFA score [20]. The retrospective design did not involve a priori sample size calculation, but post hoc considerations were informed by our experience and assessment. Sample size planning, crucial for training machine learning algorithms, employed a multiple regression analysis with a maximum of 7 variables in the final model (trajectory of SOFA Score between day 1 to 7). Assuming an 80% power, 5% significance level, and a Cohen’s f2 effect size of 0.35, a sample size of up to 68 septic patients is considered appropriate, as determined using R version 4.3.1 (R Core Team, 2023) and the “PWR” package version 1.3–0 (Champely, 2020). While machine learning methods typically demand a larger sample size than linear regression, we consider our sample size of 252 septic patients to be adequate.

The statistical analyses as well as the learning, testing and validation of the ML algorithms were performed using the software R (R version 3.5.3; The R Foundation for Statistical Computing; http://www.R-project.org). A two-sided p-value < 0.05 was considered statistically significant. The CIs were calculated with 95% coverage.

## Results

### Cohort description

A total of 252 septic patients from seven ICUs were included in our study for final analysis (Fig 1, S1 Table). The cohort consisted of 160 male patients (64%) and the mean age was 65 (± 14) years. The median SOFA score at study inclusion was 8 (IQR: 5–11). The independent validation cohort consisted of 1,790 patients admitted to the ICU of the Amsterdam UMC. Males comprised 1085 of 1790 subjects (60%) and 58% (1041 of 1790) of all patients were aged between 50 and 79 years. An overview of further baseline characteristics is provided in Table 1 and S1 Table.

**Table 1:** Base characteristics of the derivation and validation cohort.

	Derivation cohort (n = 252)		Validation cohort (n = 1790)	
Age *yrs*. (IQR)	66	(56; 76)	n/a*	
• 18–39 *yrs*., n (%)	19	(8)	306	(17)
• 40–49 *yrs*., n (%)	16	(6)	233	(13)
• 50–59 *yrs*., n (%)	47	(19)	322	(18)
• 60–69 *yrs*., n (%)	69	(27)	393	(22)
• 70–79 *yrs*., n (%)	61	(24)	326	(18)
• 80+ *yrs*., n (%)	40	(16)	214	(12)
Male sex, n (%)	160	(64)	1085	(60)
Admission SOFA Score (IQR)	8	(5; 11)	8	(5; 10)
• Central nervous system	1	(0; 4)	2	(0; 4)
• Renal	1	(0; 3)	0	(0; 0)
• Liver	0	(0; 0)	0	(0; 0)
• Coagulation	0	(0; 0)	0	(0; 1)
• Cardiovascular	3	(0; 4)	4	(1; 4)
• Respiratory	2	(1; 3)	2	1; 3)
Mechanical ventilation, n (%)	186	(90)	1618	(89)
ICU length of stay, *days* (IQR)	7.5	(2.9; 14.9)	3.9	(1.8; 9.4)
Comorbid condition, n (%)				
• Alcoholism	16	(9)		
• Hypertension	123	(70)		
• Chronic kidney disease	40	(23)		
• COPD	26	(15)		
• Other lung disease	17	(10)		
• Diabetes mellitus	53	(30)		
• Obesity	49	(28)		
• Cardiovascular disease	69	(39)		
• Malignancy	40	(23)		
• Nicotine dependence	38	(22)		
• Dialysis	10	(6)		
• Organ transplantation	27	(15)		
Laboratory value, *day 1* (IQR)				
• C-reactive protein, mg/dl	16.3	(9.3; 26.6)		
• Procalcitonin, ng/ml	3.0	(0.5; 12:3)		
• Lactate, mmol/l	1.4	(1.9; 2.2)		
• White blood cells, n/μl	14	(9; 19)		
30-day mortality, n (%)	76	(30)	462	(26)

Data are presented as n (%); median, IQR (25^th^, 75^th^ percentile)

*Due to data protection laws, age is not accessible in the Amsterdam UMC database

### Performance of ΔSOFA score for the prediction of mortality in the derivation cohort

We generated a ROC for the derivation cohort (Fig 2) to validate the ΔSOFA algorithm proposed by Karakike et al. [6]. Here, the ΔSOFA score between day 1 and 7 (ΔSOFA7) yielded an AUROC of 0.727 (95% CI: 0.654–0.800) (Table 2). The AUROCs for shorter time periods were 0.695 (95% CI: 0.620–0.770) for Δ of day 1 to day 5 (ΔSOFA5) and 0.661 (95% CI: 0.584–0.738) for Δ of day 1 to 3 (ΔSOFA3) (Table 2). As a side note, the isolated examination of the SOFA score on the first day yielded an AUROC of 0.76 (95%-CI: 0.70–0.82) for predicting 30-day mortality.

**Table 2:** Areas under the receiver operator characteristic curve (AUROC) of the ΔSOFA score and the machine learning (ML) classifier models for the derivation and validation cohort.

		Derivation cohort			Validation cohort		
	**Algorithm**	**AUROC**	**95 % CI**	**Statistically different to ΔSOFA**	**AUROC**	**95 % CI**	**Statistically different to derivation cohort**
**Day 1 to 7**	ΔSOFA	0.727	0.654–0.800	N/A	0.689	0.659–0.719	p = 0.34
	SVM with polynomial Kernel	0.837	0.712–0.963	p = 0.02*	0.819	0.768–0.871	p = 0.59
	aNN	0.822	0.692–0.951	p = 0.05*	0.817	0.766–0.869	p = 0.88
**Day 1 to 5**	ΔSOFA	0.695	0.620–0.770	N/A	0.673	0.643–0.703	p = 0.59
	SVM with polynomial Kernel	0.824	0.694–0.953	p < 0.01*	0.798	0.744–0.851	p = 0.45
	aNN	0.801	0.666–0.937	p = 0.04*	0.799	0.745–0.852	p = 0.96
**Day 1 to 3**	ΔSOFA	0.661	0.584 – 0.738	N/A	0.642	0.612–0.672	p = 0.65
	SVM with polynomial kernel	0.816	0.684–0.947	p < 0.01*	0.776	0.721–0.832	p = 0.25
	aNN	0.797	0.661–0.933	p < 0.01*	0.766	0.710–0.823	p = 0.39

Both ML algorithms outperformed the ΔSOFA score for all time spans tested. All results could be reproduced in the validation cohort. Support Vector Machine: SVM, artificial neural network: aNN.

### Machine learning algorithms’ performance in the derivation cohort

We trained and tested seven ML algorithms for three time spans. We selected an aNN and a SVM with polynomial kernel for further analysis. Both ML algorithms showed significantly higher AUROCs over seven days than the ΔSOFA7 score. On the other hand, there was no significant difference between the two ML algorithms with the aNN showing an AUROC of 0.822 (95% CI: 0.692–0.951) and the SVM algorithm yielding 0.837 as AUROC (95% CI: 0.712–0.963; Table 2). The predictive power of the ML algorithms for shorter timespans remained stable (AUROC of the SVM was 0.824 for five days and 0.816 for three days; Table 2). Both ML algorithms significantly outperformed the ΔSOFA7, ΔSOFA5 and ΔSOFA3 for all three time-intervals assessed (Table 2). The performance results of all ML-based models are provided as S2 Table. An insight into the feature importance of the aNN and SVM models, as well as two example cases with a breakdown of the prediction process, can be found in S3 File. Sensitivity and specificity regarding the performance of the aNN and SVM models can be found in S3 Table.

### Comparison and performance of the machine learning classification models in the validation cohort

Both ML algorithms reproduced similar AUROCs in the validation cohort compared to the derivation cohort for seven days (aNN: AUROC = 0.817, p = 0.88, SVM: AUROC = 0.819, p = 0.59, Table 3). Equivalently, AUROCs of 0.799 and 798 (aNN and SVM) and 0.766 and 0.776 (aNN and SVM) were shown for five days and three days, respectively. In like manner, the ΔSOFA algorithm also showed similar AUROCs in the validation cohort to the derivation cohort (AUROC = 0.689, p = 0.34 at day 7, 0.673, p = 0.59 at day 5 and 0.642, p = 0.65 at day 3; Table 2.)

**Table 3:** The AUROC, the net reclassification improvement (NRI) and the Integrated Discrimination Index (IDI) of the Δ SOFA approach vs. aNN are shown.

		Values	95 % CI	p-value
Day 1 to 7	AUROC			
	Δ SOFA 7d	0.727	(0.654–0.800)	N/A
	aNN 7d	0.822	(0.692–0.951)	p = 0.05
	NRI			
	Category-free NRI (%)	30.6	(17.8–43.2)	p < 0.01
	% of survivors correctly reclassified	26.6		
	% of deceased correctly reclassified	4.0		
	IDI	0.361	(0.244–0.483)	p < 0.01
Day 1 to 5	AUROC			
	Δ SOFA 5d	0.695	(0.620–0.770)	N/A
	aNN 5d	0.801	(0.666–0.937)	p = 0.04
	NRI			
	Category-free NRI (%)	21.5	(7.4–34.6)	p < 0.01
	• % of survivors correctly reclassified	32.2		
	• % of deceased correctly reclassified	-10.7		
	IDI	0.244	(0.127–0.358)	p < 0.01
Day 1 to 3	AUROC			
	Δ SOFA 3d	0.661	(0.584–0.738)	N/A
	aNN 3d	0.797	(0.661–0.933)	p < 0.01
	NRI			
	Category-free NRI (%)	29.6	(16.2–42.4)	p < 0.01
	• % of survivors correctly reclassified	39.0		
	• % of deceased correctly reclassified	-9.4		
	IDI	0.302	(0.188–0.407)	p < 0.01

The NRI is further divided into the proportions of patients who were correctly reclassified as survivors or deceased. The aNN showed a significant improvement in prediction for all time periods considered. This was achieved predominantly by obtaining a better discrimination of survivors using the ML algorithm.

### Performance of ΔSOFA compared to artificial neural networks regarding prediction accuracy

Regarding possible improvements in patient discrimination, we compared the ΔSOFA to the aNN in further detail. Considering the NRI and integrated IDI, we observed a significant improvement in the prediction accuracy for all time periods observed. The aNN7 versus ΔSOFA7 showed a category-free NRI of 30.6%, resulting in the correct reclassification of nearly 1/3 of patients via ML (NRI 30.6%, 95% CI (17.8–43.2%), p < 0.01, IDI 0.361, p < 0.01). This significant improvement was reproduced for the time period between day 1 and 5 (NRI 21.5%, 95% CI (7.4–34.6%), p < 0.01, IDI 0.244, p < 0.01), and day 1 and 3 (NRI 29.6%, 95% CI (16.2–42.4%), p < 0.01, IDI 0.302, p < 0.01). A further subset of the NRI is provided in Table 3.

**Fig 3:**
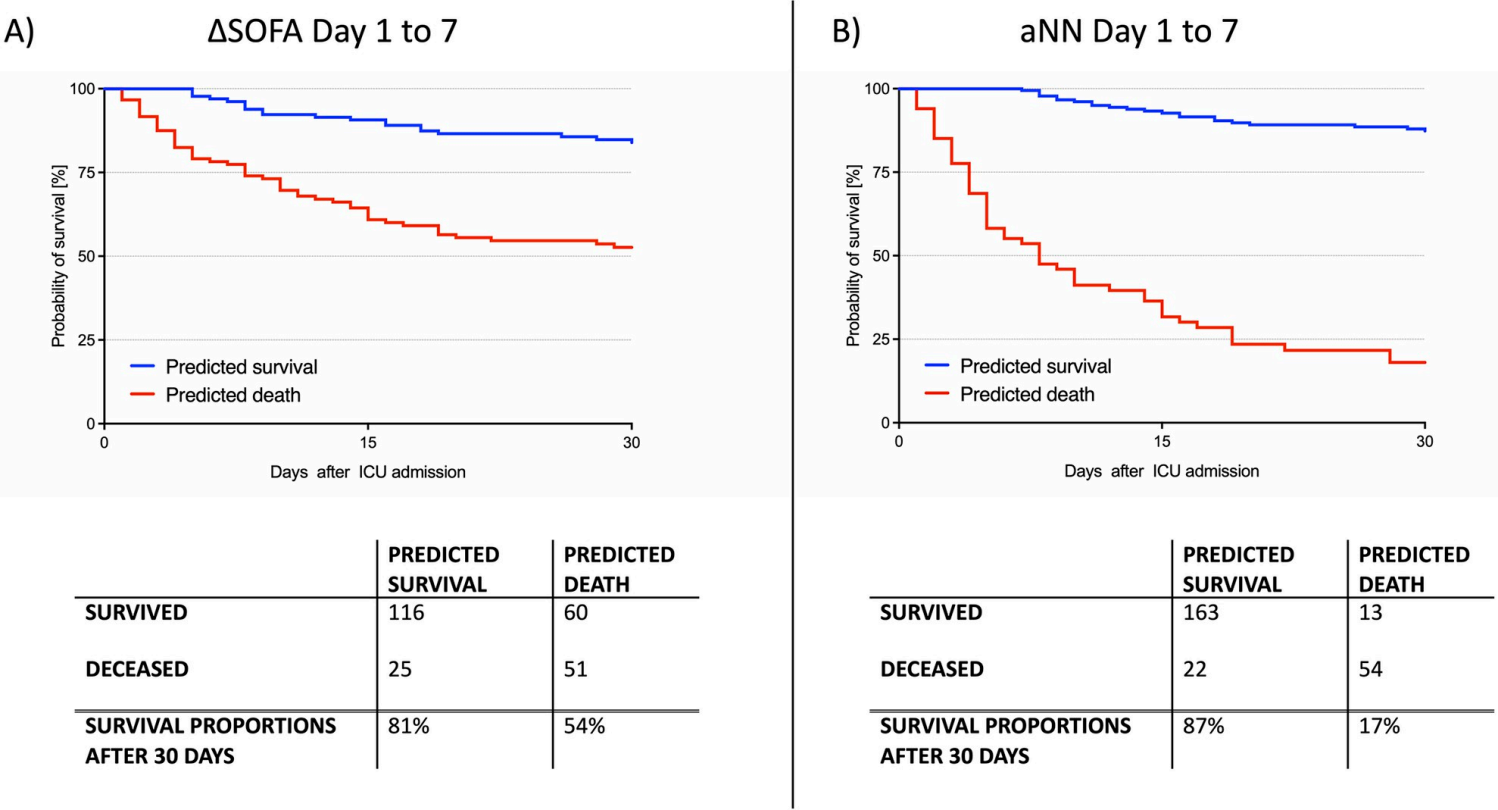
Kaplan Meier curves, cross-tabulations and the proportion of patients who survived for the a) ΔSOFA score (day 1 and 7, cutoff via Youden Index = 0.35) and b) artificial neural network (aNN) (day 1 till 7). Both models showed an adequate performance in detecting survivors. However, the ΔSOFA score incorrectly predicted death within 30 days for a high number of cases. A total of 54% of the patients who were predicted as deceased according to ΔSOFA score was still alive after 30 days. This percentage could be reduced to 18% by using the artificial neural network.

Analysis of the Kaplan-Meier curves confirmed the improvement, particularly regarding the prediction of survivors. Whereas according to ΔSOFA7, 111 of 252 cases were expected to die within 30 days, in fact, 176 patients were still alive at that time point. This corresponds to a proportion of 54% of the cases in which death was incorrectly estimated. Using the aNN, mortality could be predicted significantly more accurately, with only 17% of all cases predicted to die still alive after 30 days (Fig 3). Kaplan Meier curves for ΔSOFA5 vs. aNN5 and ΔSOFA3 vs. aNN3 are available in S4 File.

## Discussion

The main findings of this study are that: 1) ML-based algorithms outperformed the accuracy regarding 30-day mortality prediction compared to the conventional ΔSOFA approach when assessing daily SOFA score measurements. This advantage was even more evident when shorter time intervals were applied (i.e. ICU day 1 to 3). 2) ML-based algorithms achieved a comparably robust performance in the independent external validation cohort in contrast to the ΔSOFA, suggesting an easier generalizability and translatability.

### Using ML algorithms for the prediction of patient prognosis

With the progress of digitalization, a wide range of patient-related information has been collected on ICUs over time, generating clinical big data considering the entire length of the ICU stay. These longitudinal data seem particularly well suited to describe the individual course of a complex disease such as sepsis, thus, providing an important aspect towards precision medicine in critically ill patients [21]. Here, recent research has clearly demonstrated that the inclusion of multiple variables into ML-based prediction models in a complex and dynamical disease such as sepsis had significant advantages, although several of the variables were either difficult to analyze or not routinely used [22].

In this regard, building robust prediction models for general purpose has often previously been neglected in favor of maximizing prognostic accuracy by implementing an intricate set of input variables. Consequently, although ML and artificial intelligence for assessing complex patient data has been available for years, the translation into new prediction-support tools, useable in clinical routine, is generally lacking. A major problem is the limited reproducibility in an independent cohort, which hinders generalizability and translation. The high extent of missing variables in the validation datasets, due to implementing uncommon and rare sets of input variables, makes broad application across different ICU settings especially difficult. In addition, physicians lack confidence in algorithms that use large, complicated datasets due to the lack of comprehensibility [23, 24]. However, a certain level of trust in ML-based models by potential users is an important factor that is often underestimated [25]. Our approach holds the advantage that users merely need to input a clinically established variable–the SOFA Score–into the ML model. The simplicity of this process ensures a certain degree of traceability, given the low dimensionality and clarity of our input data. Consequently, we contend that simpler AI approaches will gain greater acceptability and face fewer justifiable concerns.

Despite the digital age of medicine, the 25-year-old SOFA is still a key element in the diagnosis and assessment of the severity of sepsis worldwide, without the need for technological support [26]. Consequently, recent research groups also aimed to adopt the development of ML-based algorithms into a more pragmatic approach and limited the input variables, for example, to the six components of the SOFA score on day 1 [27]. By analyzing these variables using artificial intelligence, their results yielded a promising prediction of 30-day mortality, and these results were still understandable for most ICU physicians, thus, supporting their clinical acceptance. We also deliberately chose a pragmatic approach in our study to minimize the potential skepticism and did not enforce maximizing prognostic enrichment by using only the daily SOFA scores as an input variable. Indeed, the ML-based algorithm outperformed the accuracy regarding 30-day mortality prediction compared to the conventional ΔSOFA without requiring additional data input.

This superior performance of ML-based models is partially attributable to their ability to learn the latent structure and complex relationships even from simple input datasets. This includes temporary trends and undulations even from low-level input data, such as in our study, solely using daily SOFA score measurements. Due to their internal ‘memory’ mechanism, ML-based mortality prediction based on time series of the SOFA score can learn and handle these dynamic and complex temporal patterns [28]. Such crucial information is neglected in conventional models, especially when assessing only the boundary values (i.e. start and end day) of a time interval, as it is the case in ΔSOFA.

### The benefit of ML algorithms compared to ΔSOFA in clinical application

Reproducibility and generalizability is of relevance in the development of suitable tools for translation into clinical routine. We validated the ML-based algorithms and ΔSOFA in an independent external cohort; it resulted in no statistically significant difference to the derivation cohort. In addition to the outperformance of the ML algorithms regarding the AUC, especially the lack of ability in detecting survivors of ΔSOFA (i.e. low specificity) remained. Accordingly, the issue concerning the reproducibility of Karakike et al.’s approach, as evident in their own validation cohort, also became apparent in both of our cohorts, questioning the widespread usability of the ΔSOFA approach for mortality prediction [6]. Our models were trained in a derivation cohort with a very low density of missing values (< 1%), which rarely occurs in the normal clinical setting. Strikingly, the ML models still showed robust performance in the validation cohort with higher frequency of missing values (approx. 8%). This indicates that our ML algorithms were indeed able to handle this higher degree of missing values by using our simple approach (last SOFA score carried forward) in a regular clinical setting.

In addition to the reliability of the test, the time from diagnosis until robust prediction results are available is of particular importance. It is desirable to receive a reliable feedback as early as possible because the initiation of sepsis therapy is time-critical and contributes significantly to the outcome [29]. However, the time period between day 1 and 7 needed for the ΔSOFA score in critically ill patients is rather long to provide prognostic information on patient outcome and, therefore, impractical. The ML appeared to be well suited for the evaluation of longitudinal data [30] and previous studies showed that including SOFA score measurements beyond day 5 of sepsis patients on ICU did not improve the accuracy to predict mortality when creating multivariate models [31]. Therefore, we also assessed whether shorter time spans also lead to robust results when using our ML approaches. Strikingly, the ML algorithms still yielded sufficiently high AUROCs for five days and even for three days, which could also be successfully reproduced in the validation cohort. By contrast, the ΔSOFA score performed significantly worse for shorter time spans in our data, as supported in the literature [6]. Impressively, ML algorithms demonstrated a more robust prediction of 30-day mortality within three days from diagnosis compared to the ΔSOFA score over a seven-day period. Regarding this important aspect, a useful tool has been created with relevant clinical benefits, since the physician can receive feedback on his/her therapy within a reduced period of time. With further research and validation, approaches like ours, uniting manageable datasets with the advanced opportunities of ML, could update the dynamic mortality prediction of sepsis patients in real time. These models with simplified input do not have to be limited solely to the SOFA score, but can be extended with other simple parameters, such as serum lactate concentration and age, to further improve their predictive accuracy as other studies indicated [32]. A convincing advantage of this pragmatic approach is that these ‘simple’ ML algorithms can, even now, be easily integrated into the existing ICIS systems on ICUs. However, an important aspect that needs to be considered, is that the application of such ML-based support tools in clinical routine is accompanied by significant regulations in many countries. At least in Europe, there is a significant regulatory overhead, as ML-based support tools must adhere to the European Medical Devices Regulation. This important regulatory aspect must be addressed before such a ML-based support tools are allowed be implemented in clinical routine.

The ML-based algorithms and the ΔSOFA approach showed comparable probability estimations regarding predicting 30-day survival in our cohort of septic patients, however, ΔSOFA significantly overestimated the probability of death, as evident from Kaplan Meier curves and NRI. The magnitude of error aside, the latter could contribute to a scenario in which clinicians suffer from alarm fatigue and fail to identify critically ill patients, which is a major concern regarding automatized early warning systems on ICUs [33]. The ML-based algorithms can be easily automated, thus, they are also well suited to capitalize on the emerging demand and availability of electronic health record data.

In this context, our findings illuminate the potential of ML-based prediction models to enhance clinical decision-making by serving as early-warning systems. In accordance with recent literature, we are confident that these intelligent support tools can empower attending physicians to allocate their attention more effectively to patients at the highest risk, thereby contributing to improved outcomes. Previous studies have compellingly demonstrated that ML-based algorithms designed for sepsis prediction significantly enhance outcomes by prompting early vigilance among attending physicians for high-risk patients [34]. Applied to our work, this capability could facilitate a focused allocation of attention to patients requiring therapeutic or diagnostic interventions, thereby alleviating the risk of crucial information being overlooked—particularly in the data-dense environment of the ICU.

Another interesting finding is that the performance of the individual ML algorithms showed minor differences. Therefore, based on our data and the pragmatic approach, complex algorithms are not inevitably beneficial, since simple algorithms such as logistic regression also delivered stable performance (S2 Table).

### Limitations

A limitation of the present study is that that the number of patients in our derivation cohort, specifically for training ML models, is relatively small. In our work, we deliberately chose the approach of using the smaller, prospective cohort to train the ML models because of the confirmed sepsis diagnosis, the high number of complete datasets and confirmed follow-up. The potential bias from overfitting the models was weighed against the potential selection bias in a retrospective cohort, which is a common issue particularly in sepsis [35]. Since we used rather simple input variables (i.e., SOFA score 1–7) and an independent validation cohort to detect overfitting, we decided to train on the high quality but smaller dataset.

A potential bias in our ML models is the fact that a number of patients have deceased during the time periods considered (i.e., 7, 5, and 3 days), which may have affected the learning effect. Even if less than half of all deaths among the derivation cohort occurred within the first 7 days (S4 Table), after analysis of the feature importance a balanced ratio of the variables within the models is present (S3 File) and the AUROC could be reproduced in the independent validation cohort, it cannot be completely ruled out that this may have limited the performance of our models.

Moreover, whereas the validation of 30-day survival prediction was largely consistent between the retrospective and prospective datasets, there was more variability concerning some baseline parameters that should not be neglected. A possible reason for this might be structural differences between the retro- and prospective datasets, for example, changes in treatment or age cohort over time and different regional treatment patterns. Strikingly, our trained ML models still performed robustly in the validation cohort even when considering potential temporal changes (S5 Table). Thus, the strength of our approach is the generalizability across institutions and even other similarly resourced countries.

While our study primarily centers on developing and evaluating mortality predictive models, we recognize the importance of investigating their real-world impact on clinical decision-making and patient care. Accurate mortality prediction may have the potential to guide timely interventions, personalized treatments, and resource optimization, potentially improving patient outcomes. However, the direct translation of mortality prediction to enhanced patient care requires additional exploration, including prospective studies and clinical trials, to establish a clear causal link.

### Conclusion

Our results demonstrate that ML-based algorithms assessing the daily dynamics of the SOFA scores are superior to the conventional ΔSOFA score when predicting the 30-day mortality in critically ill adult septic patients in the ICU. Therefore, this approach could provide a promising and automated support tool with intelligent mechanisms for the standardized assessment of the individual disease course using daily SOFA score measurements. These ML-based algorithms could be easily implemented in electronic health record systems, therefore, they offer a valuable support tool, especially for ICU physicians.

## Supporting information

S1 File Distribution of AUC within the cross validation with mean AUC for each ML model. (PDF)

S2 File Access to ML models. (PDF)

S3 File Feature importance of the aNN and SVM models and two example cases with a breakdown of the prediction process. A-C) Analysis of Feature Importance for the aNN and SVM models (each for 3, 5, or 7 days input after sepsis diagnosis). Based on permutations, the contribution of individual features (i.e., the SOFA Scores of the respective days) to the model’s AUC was examined. Using the example of the aNN for 7 days, it is evident that the input variable "SOFA Score on Day 6" is crucial for the final AUC of the model. If this variable is replaced by random permutations, rendering it informationless, the model’s AUC would deteriorate by 0.322. D-E) Analysis of Shapley Values in 2 example cases. This illustrates the proportion of each input variable (i.e., the respective SOFA Scores) in determining the final prediction outcome.
(PDF)

S4 File Kaplan Meier curves, cross tables and proportion of survived patients for the a) ΔSOFA score and b) artificial neural network (aNN). 1: Day 1–5. 2: Day 1–3.
(PDF)

S1 Table Dataset for training ML models (SepsisDataNet.NRW Cohort). (XLSX)

S2 Table Performance of ΔSOFA and machine learning algorithms in primary (training) cohort and validation cohort. (DOCX)

S3 Table Performance of aNN and SVM models focusing on sensitivity and specificity. a) Aiming for a sensitivity of at least 90% while maximizing specificity b) Aiming for a specificity of at least 90% while maximizing sensitivity.
(DOCX)

S4 Table Breakdown of mortality within the first seven days and at the end of 30-day follow-up. (DOCX)

S5 Table Performance of ΔSOFA and machine learning algorithms in validation cohort stratified by admission intervals. (DOCX)

S1 Data (ZIP)

## List of abbreviations

AUROC: area under the receiver operator characteristic curve
aNN: artificial neural network
CI: confidence intervals
ICU: intensive care units
IDI: integrated discrimination improvement
ML: Machine learning
NRI: net reclassification improvement
ROC: receiver operator characteristic
SOFA: sequential organ failure assessment
SVM: support vector machine

## References

[1] Raith E.P., et al., Prognostic Accuracy of the SOFA Score, SIRS Criteria, and qSOFA Score for In-Hospital Mortality Among Adults With Suspected Infection Admitted to the Intensive Care Unit. JAMA, 2017. 317(3): p. 290–300. doi: 10.1001/jama.2016.20328 28114553

[2] de Grooth H.J., et al., SOFA and mortality endpoints in randomized controlled trials: a systematic review and meta-regression analysis. Crit Care, 2017. 21(1): p. 38. doi: 10.1186/s13054-017-1609-1 28231816 PMC5324238

[3] Seymour C.W., et al., Assessment of Clinical Criteria for Sepsis: For the Third International Consensus Definitions for Sepsis and Septic Shock (Sepsis-3). JAMA, 2016. 315(8): p. 762–74. doi: 10.1001/jama.2016.0288 26903335 PMC5433435

[4] Ferreira F.L., et al., Serial evaluation of the SOFA score to predict outcome in critically ill patients. JAMA, 2001. 286(14): p. 1754–8. doi: 10.1001/jama.286.14.1754 11594901

[5] Agency E.M., Guideline on clinical investigation of medicinal products for the treatment of sepsis. CHMP/EWP/4713/03 2006.

[6] Karakike E., et al., The early change of SOFA score as a prognostic marker of 28-day sepsis mortality: analysis through a derivation and a validation cohort. Crit Care, 2019. 23(1): p. 387. doi: 10.1186/s13054-019-2665-5 31783881 PMC6884794

[7] Minne L., Abu-Hanna A., and de Jonge E., Evaluation of SOFA-based models for predicting mortality in the ICU: A systematic review. Crit Care, 2008. 12(6): p. R161. doi: 10.1186/cc7160 19091120 PMC2646326

[8] Soo A., et al., Describing organ dysfunction in the intensive care unit: a cohort study of 20,000 patients. Crit Care, 2019. 23(1): p. 186. doi: 10.1186/s13054-019-2459-9 31122276 PMC6533687

[9] Attia Z.I., et al., Screening for cardiac contractile dysfunction using an artificial intelligence-enabled electrocardiogram. Nat Med, 2019. 25(1): p. 70–74. doi: 10.1038/s41591-018-0240-2 30617318

[10] Chen T., et al., Prediction and Risk Stratification of Kidney Outcomes in IgA Nephropathy. Am J Kidney Dis, 2019. 74(3): p. 300–309. doi: 10.1053/j.ajkd.2019.02.016 31031086

[11] He J., et al., The practical implementation of artificial intelligence technologies in medicine. Nat Med, 2019. 25(1): p. 30–36. doi: 10.1038/s41591-018-0307-0 30617336 PMC6995276

[12] Allam A., et al., Analyzing Patient Trajectories With Artificial Intelligence. J Med Internet Res, 2021. 23(12): p. e29812. doi: 10.2196/29812 34870606 PMC8686456

[13] Tseng P.Y., et al., Prediction of the development of acute kidney injury following cardiac surgery by machine learning. Crit Care, 2020. 24(1): p. 478. doi: 10.1186/s13054-020-03179-9 32736589 PMC7395374

[14] Raita Y., et al., Emergency department triage prediction of clinical outcomes using machine learning models. Crit Care, 2019. 23(1): p. 64. doi: 10.1186/s13054-019-2351-7 30795786 PMC6387562

[15] Thoral P.J., et al., Sharing ICU Patient Data Responsibly Under the Society of Critical Care Medicine/European Society of Intensive Care Medicine Joint Data Science Collaboration: The Amsterdam University Medical Centers Database (AmsterdamUMCdb) Example. Crit Care Med, 2021. 49(6): p. e563–e577. doi: 10.1097/CCM.0000000000004916 33625129 PMC8132908

[16] Komorowski M., et al., The Artificial Intelligence Clinician learns optimal treatment strategies for sepsis in intensive care. Nat Med, 2018. 24(11): p. 1716–1720. doi: 10.1038/s41591-018-0213-5 30349085

[17] Lambden S., et al., The SOFA score-development, utility and challenges of accurate assessment in clinical trials. Crit Care, 2019. 23(1): p. 374. doi: 10.1186/s13054-019-2663-7 31775846 PMC6880479

[18] Molinaro A.M., Simon R., and Pfeiffer R.M., Prediction error estimation: a comparison of resampling methods. Bioinformatics, 2005. 21(15): p. 3301–7. doi: 10.1093/bioinformatics/bti499 15905277

[19] Hanley J.A. and McNeil B.J., The meaning and use of the area under a receiver operating characteristic (ROC) curve. Radiology, 1982. 143(1): p. 29–36. doi: 10.1148/radiology.143.1.7063747 7063747

[20] Pencina M.J., et al., Evaluating the added predictive ability of a new marker: from area under the ROC curve to reclassification and beyond. Stat Med, 2008. 27(2): p. 157–72; discussion 207–12. doi: 10.1002/sim.2929 17569110

[21] Sanchez-Pinto L.N., Luo Y., and Churpek M.M., Big Data and Data Science in Critical Care. Chest, 2018. 154(5): p. 1239–1248. doi: 10.1016/j.chest.2018.04.037 29752973 PMC6224705

[22] Hou N., et al., Predicting 30-days mortality for MIMIC-III patients with sepsis-3: a machine learning approach using XGboost. J Transl Med, 2020. 18(1): p. 462. doi: 10.1186/s12967-020-02620-5 33287854 PMC7720497

[23] Sandhu S., et al., Integrating a Machine Learning System Into Clinical Workflows: Qualitative Study. J Med Internet Res, 2020. 22(11): p. e22421. 10.2196/22421 PMC7714645 33211015

[24] Asan O., Bayrak A.E., and Choudhury A., Artificial Intelligence and Human Trust in Healthcare: Focus on Clinicians. J Med Internet Res, 2020. 22(6): p. e15154. 10.2196/15154 PMC7334754 32558657

[25] Markus A.F., Kors J.A., and Rijnbeek P.R., The role of explainability in creating trustworthy artificial intelligence for health care: A comprehensive survey of the terminology, design choices, and evaluation strategies. J Biomed Inform, 2021. 113: p. 103655. doi: 10.1016/j.jbi.2020.103655 33309898

[26] Vincent J.L., et al., Use of the SOFA score to assess the incidence of organ dysfunction/failure in intensive care units: results of a multicenter, prospective study. Working group on "sepsis-related problems" of the European Society of Intensive Care Medicine. Crit Care Med, 1998. 26(11): p. 1793–800. doi: 10.1097/00003246-199811000-00016 9824069

[27] Pan X., et al., Evaluate prognostic accuracy of SOFA component score for mortality among adults with sepsis by machine learning method. BMC Infect Dis, 2023. 23(1): p. 76. 36747139 10.1186/s12879-023-08045-x PMC9903420

[28] Goldstein B.A., et al., Opportunities and challenges in developing risk prediction models with electronic health records data: a systematic review. J Am Med Inform Assoc, 2017. 24(1): p. 198–208. doi: 10.1093/jamia/ocw042 27189013 PMC5201180

[29] Seymour C.W., et al., Time to Treatment and Mortality during Mandated Emergency Care for Sepsis. N Engl J Med, 2017. 376(23): p. 2235–2244. doi: 10.1056/NEJMoa1703058 28528569 PMC5538258

[30] Devaux A., et al., Individual dynamic prediction of clinical endpoint from large dimensional longitudinal biomarker history: a landmark approach. BMC Med Res Methodol, 2022. 22(1): p. 188. doi: 10.1186/s12874-022-01660-3 35818025 PMC9275051

[31] Holder A.L., et al., Serial Daily Organ Failure Assessment Beyond ICU Day 5 Does Not Independently Add Precision to ICU Risk-of-Death Prediction. Crit Care Med, 2017. 45(12): p. 2014–2022. doi: 10.1097/CCM.0000000000002708 28906286 PMC5693776

[32] Liu Y., et al., A time-incorporated SOFA score-based machine learning model for predicting mortality in critically ill patients: A multicenter, real-world study. Int J Med Inform, 2022. 163: p. 104776. doi: 10.1016/j.ijmedinf.2022.104776 35512625

[33] Chromik J., et al., Computational approaches to alleviate alarm fatigue in intensive care medicine: A systematic literature review. Front Digit Health, 2022. 4: p. 843747. doi: 10.3389/fdgth.2022.843747 36052315 PMC9424650

[34] Shimabukuro D.W., et al., Effect of a machine learning-based severe sepsis prediction algorithm on patient survival and hospital length of stay: a randomised clinical trial. BMJ Open Respir Res, 2017. 4(1): p. e000234. doi: 10.1136/bmjresp-2017-000234 29435343 PMC5687546

[35] Johnson A.E.W., et al., A Comparative Analysis of Sepsis Identification Methods in an Electronic Database. Crit Care Med, 2018. 46(4): p. 494–499. doi: 10.1097/CCM.0000000000002965 29303796 PMC5851804

