# Supplementary material for: Assessing SOFA score trajectories in sepsis using machine learning: A pragmatic approach to improve the accuracy of mortality prediction
Source: PLoS One. 2024 Mar 28;19(3):e0300739. doi: 10.1371/journal.pone.0300739 (PMC10977876; doi:10.1371/journal.pone.0300739)

Supplementary File:
Distribution of AUC within the cross validation with mean AUC for each ML model

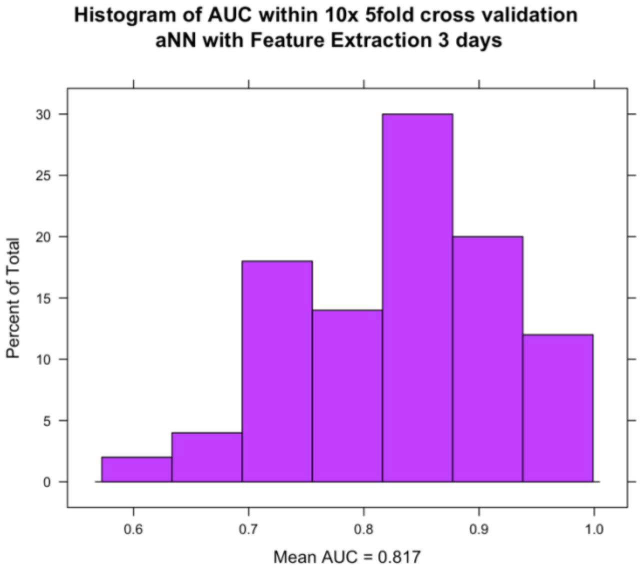

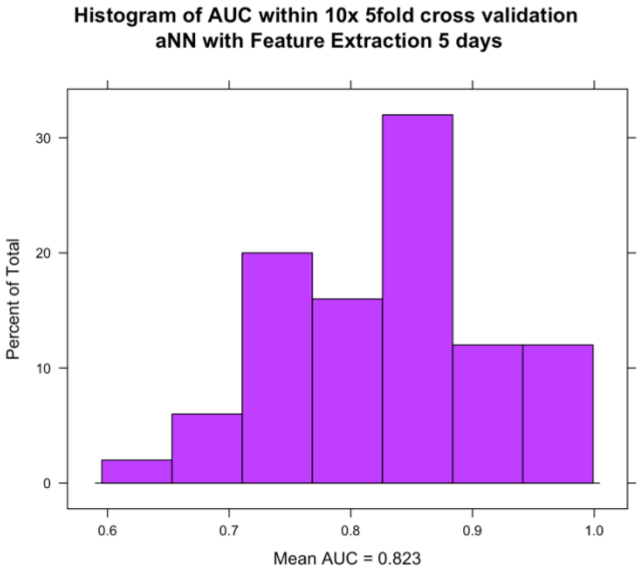

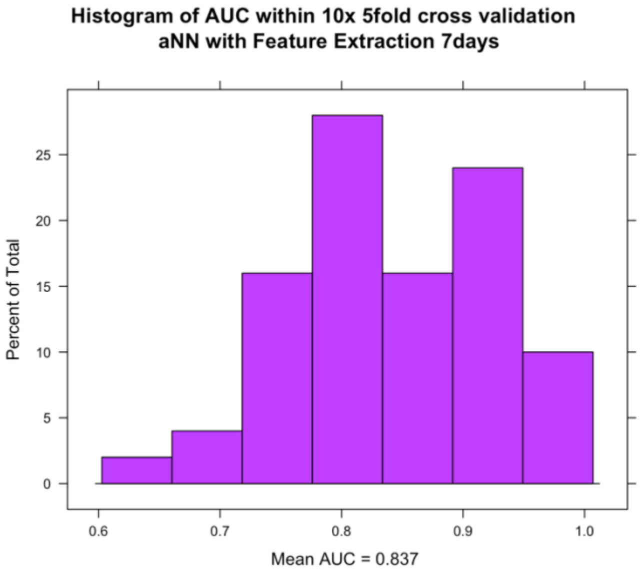

Histogram of AUC within 10x 5fold cross validation
aNN 3 days

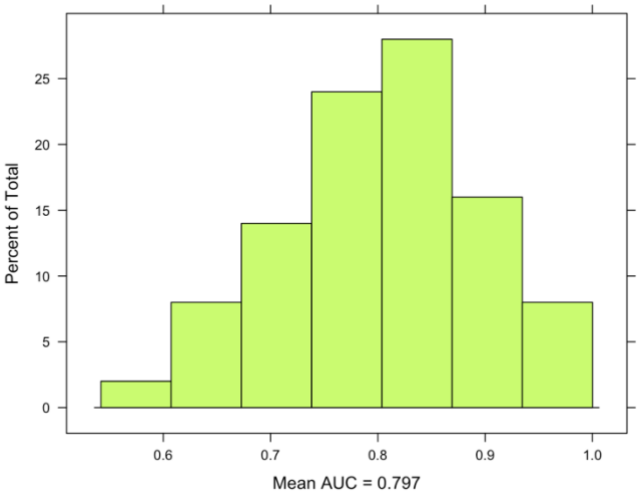

Histogram of AUC within 10x 5fold cross validation
aNN 5 days

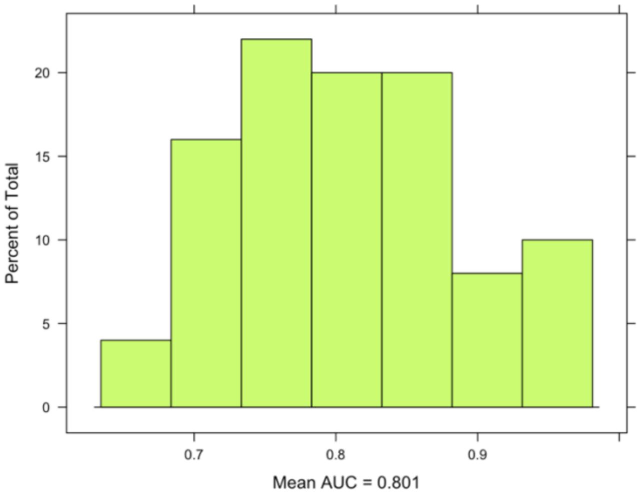

Histogram of AUC within 10x 5fold cross validation
aNN 7days

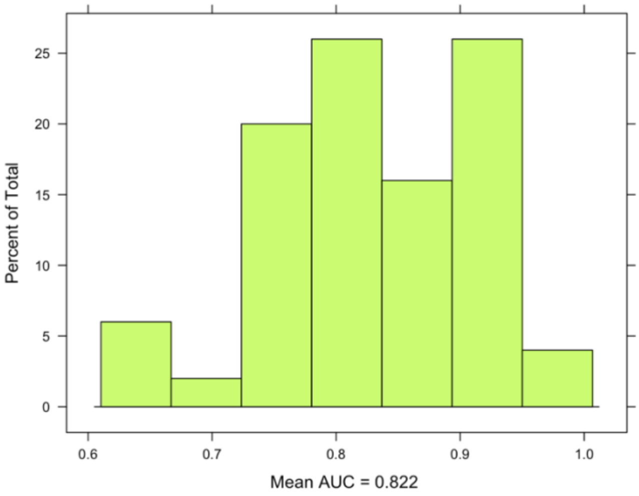

**Histogram of AUC within 10x 5fold cross validation
LDA 3 days**

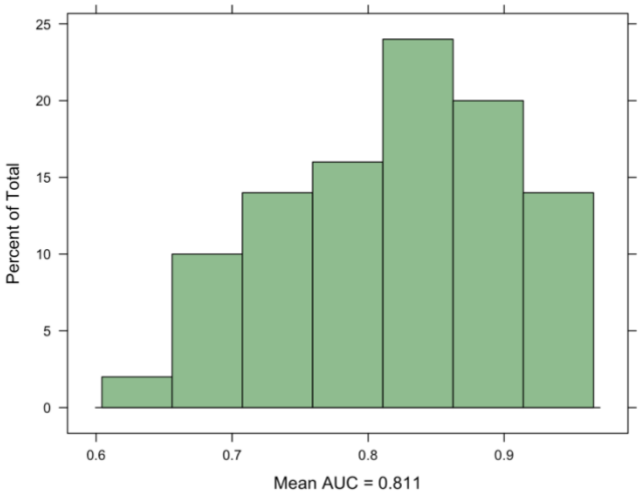

**Histogram of AUC within 10x 5fold cross validation
LDA 5 days**

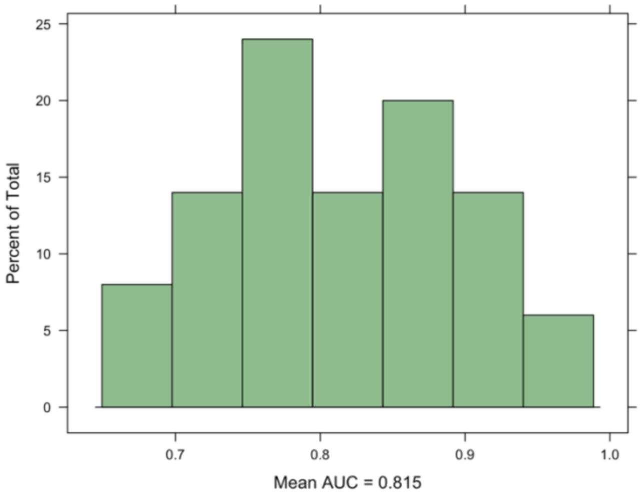

**Histogram of AUC within 10x 5fold cross validation
LDA 7 days**

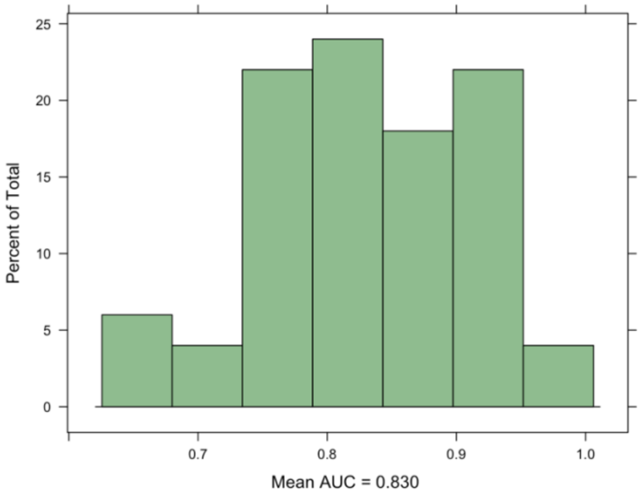

Histogram of AUC within 10x 5fold cross validation
Logistic Regression 3 days

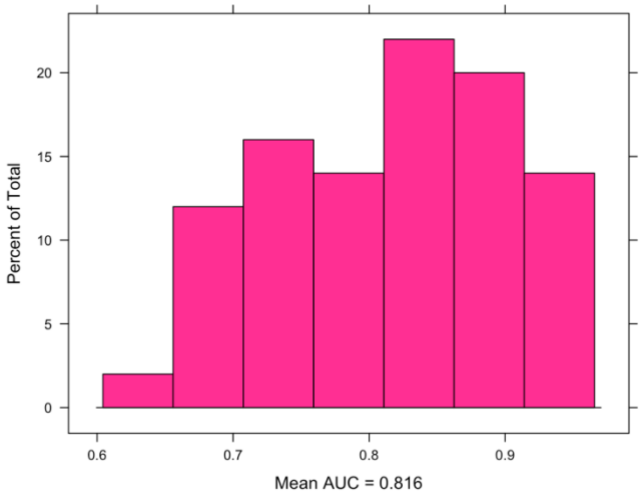

Histogram of AUC within 10x 5fold cross validation
Logistic Regression 5 days

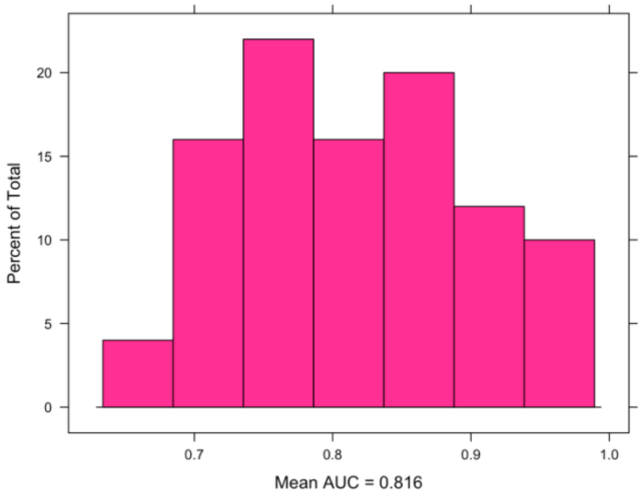

Histogram of AUC within 10x 5fold cross validation
Logistic Regression 7 days

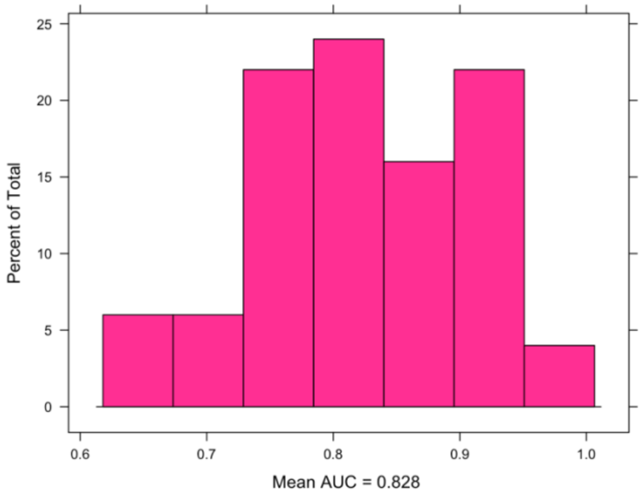

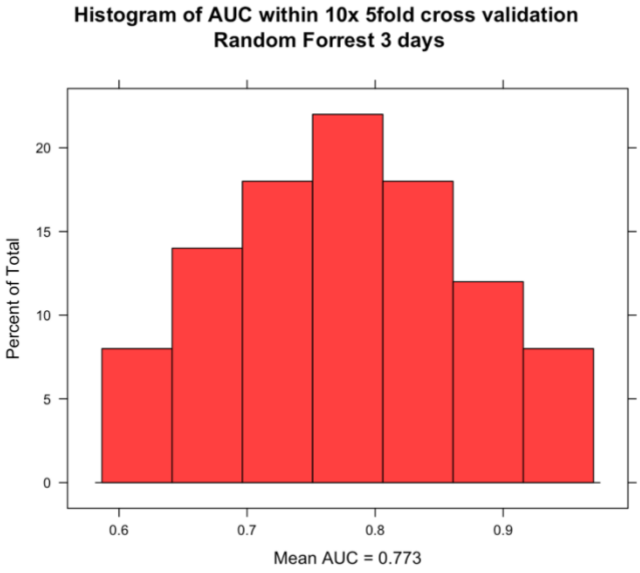
Histogram of AUC within 10x 5fold cross validation
Random Forrest 3 days
Percent of Total
0
5
10
15
20
0.6
0.7
0.8
0.9
Mean AUC = 0.773

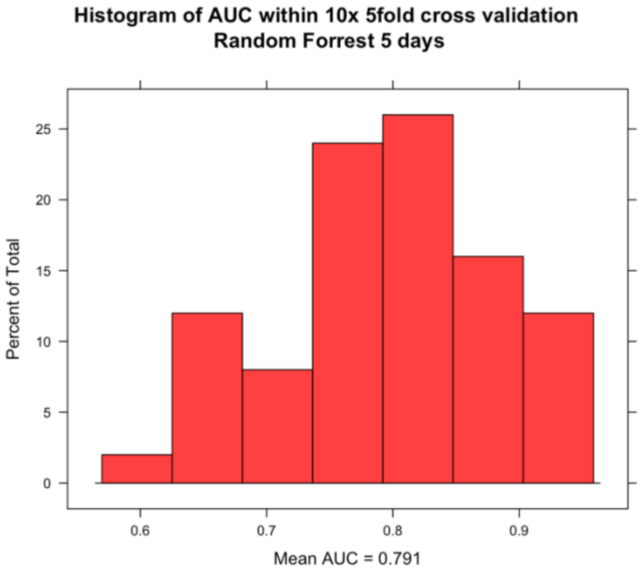
Histogram of AUC within 10x 5fold cross validation
Random Forrest 5 days
Percent of Total
0
5
10
15
20
25
0.6
0.7
0.8
0.9
Mean AUC = 0.791

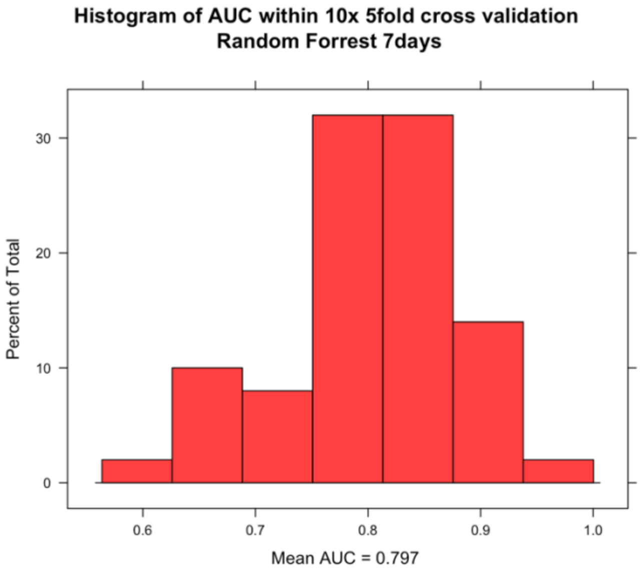
Histogram of AUC within 10x 5fold cross validation
Random Forrest 7days
Percent of Total
0
10
20
30
0.6
0.7
0.8
0.9
1.0
Mean AUC = 0.797

Histogram of AUC within 10x 5fold cross validation
SVM with polynomial kernel 3 days

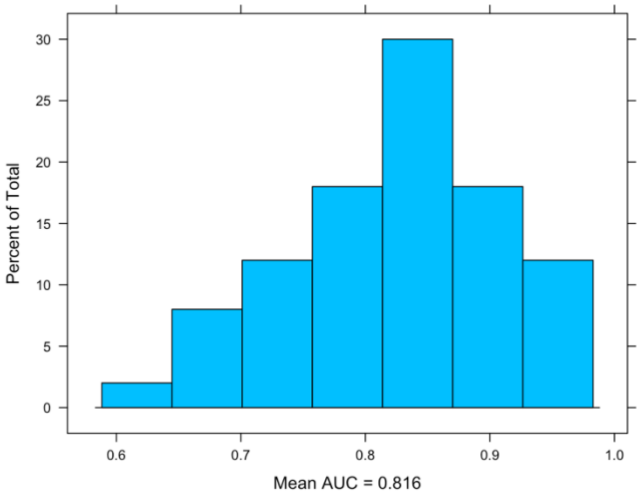

Histogram of AUC within 10x 5fold cross validation
SVM with polynomial kernel 5 days

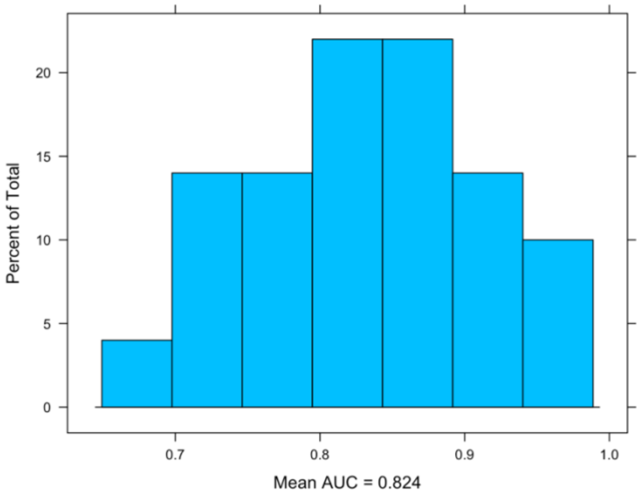

Histogram of AUC within 10x 5fold cross validation
SVM with polynomial kernel 7days

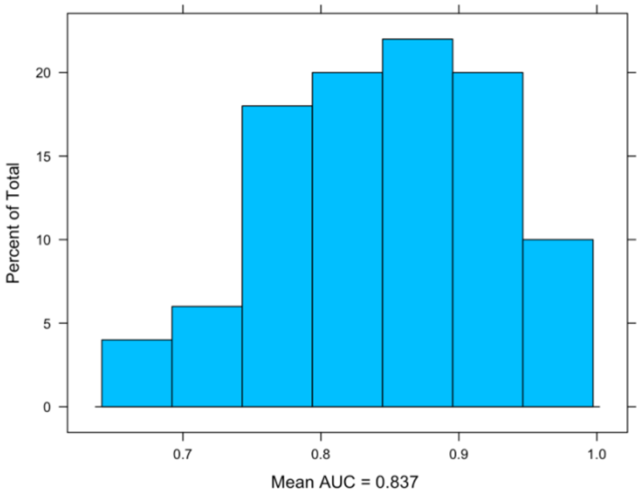

Histogram of AUC within 10x 5fold cross validation
SVM with linear kernel 3 days

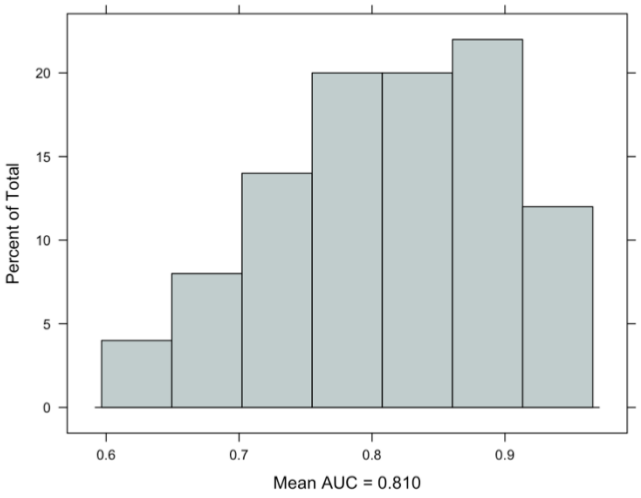

Histogram of AUC within 10x 5fold cross validation
SVM with linear kernel 5 days

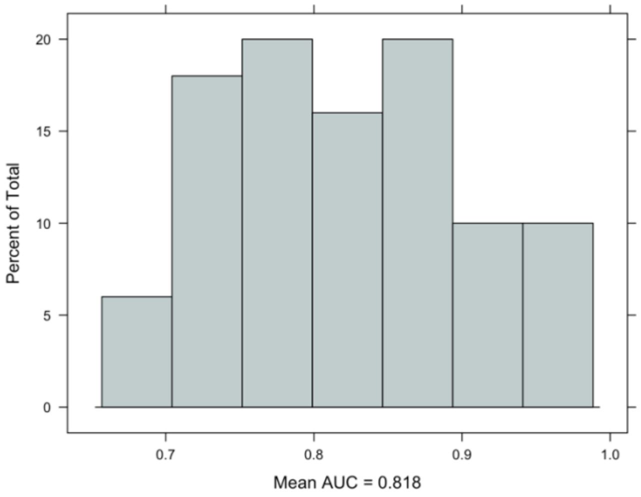

Histogram of AUC within 10x 5fold cross validation
SVM with linear kernel 7days

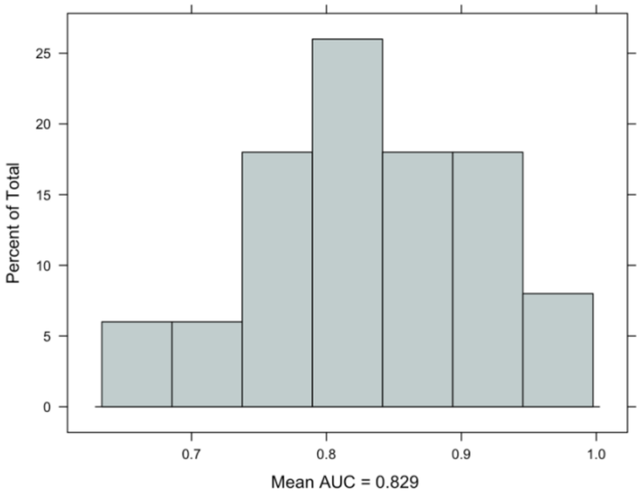

Supplement: S1 File — (PDF) [file pone.0300739.s001.pdf]
