# Supplementary material for: Assessing SOFA score trajectories in sepsis using machine learning: A pragmatic approach to improve the accuracy of mortality prediction
Source: PLoS One. 2024 Mar 28;19(3):e0300739. doi: 10.1371/journal.pone.0300739 (PMC10977876; doi:10.1371/journal.pone.0300739)
Supplement: S2 File — (PDF) [file pone.0300739.s002.pdf]

Dear readers,

all ML-models can be downloaded from the official cloud of the Ruhr-University Bochum, Germany:

https://ruhr-uni-bochum.sciebo.de/s/YJhvQXrfPCMUKEe

In addition to the models, you will also find a detailed description of how to use the models with the program "R".
