# Supplementary material for: Assessing SOFA score trajectories in sepsis using machine learning: A pragmatic approach to improve the accuracy of mortality prediction
Source: PLoS One. 2024 Mar 28;19(3):e0300739. doi: 10.1371/journal.pone.0300739 (PMC10977876; doi:10.1371/journal.pone.0300739)
Supplement: S3 File — A-C) Analysis of Feature Importance for the aNN and SVM models (each for 3, 5, or 7 days input after sepsis diagnosis). Based on permutations, the contribution of individual features (i.e., the SOFA Scores of the respective days) to the model’s AUC was examined. Using the example of the aNN for 7 days, it is evident that the input variable "SOFA Score on Day 6" is crucial for the final AUC of the model. If this variable is replaced by random permutations, rendering it informationless, the model’s AUC would deteriorate by 0.322. D-E) Analysis of Shapley Values in 2 example cases. This illustrates the proportion of each input variable (i.e., the respective SOFA Scores) in determining the final prediction outcome. (PDF) [file pone.0300739.s003.pdf]

A)

Permutation-based Feature Importance[1] for aNN 7d and SVM 7d

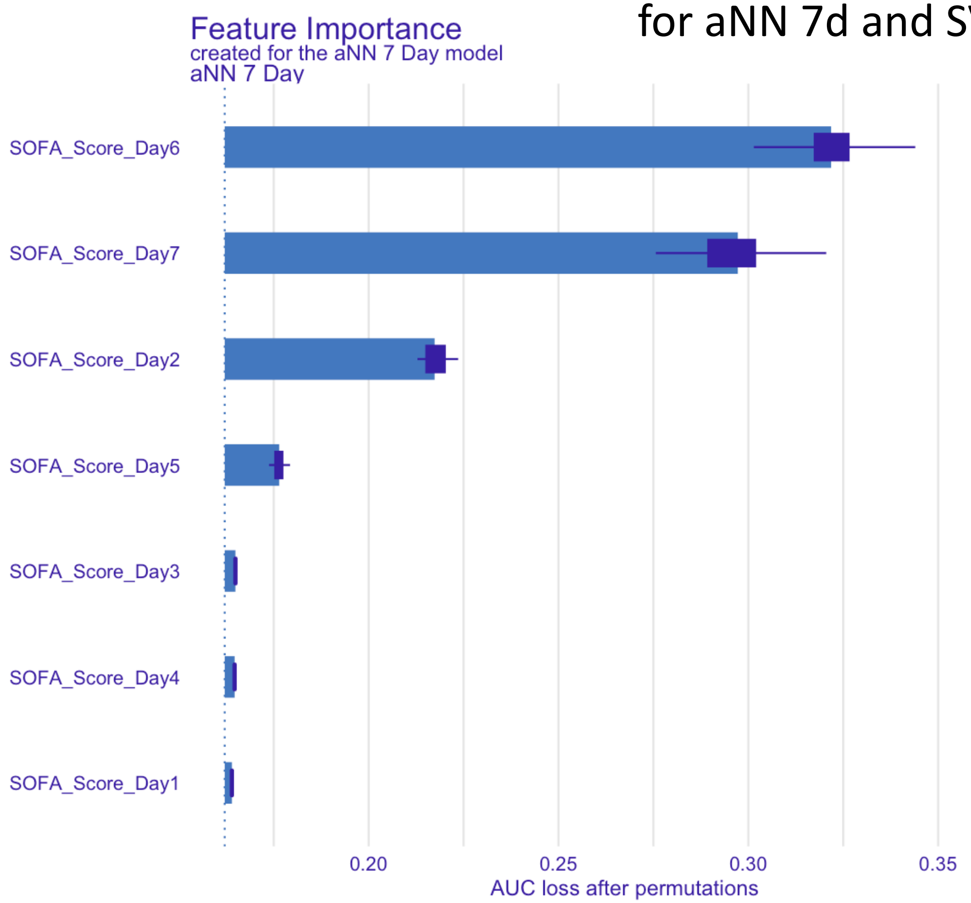

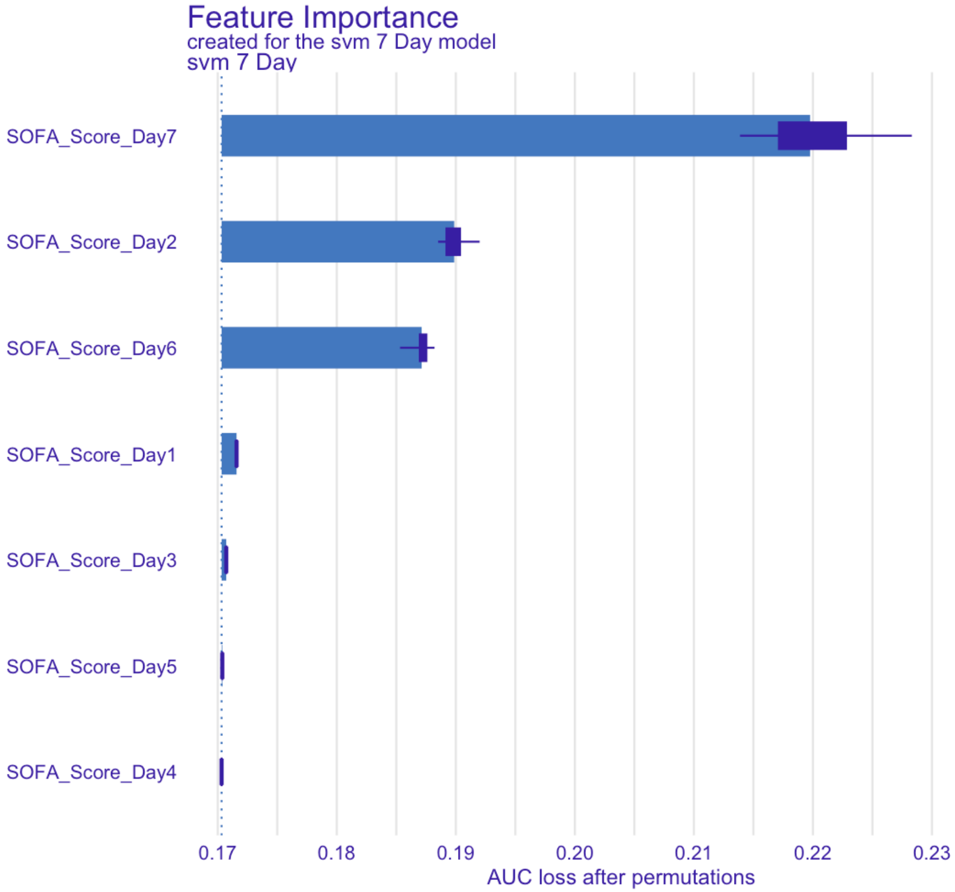

Variable in aNN7 Day Model	Mean AUC loss
SOFA Score Day 1	0.164
SOFA Score Day 2	0.217
SOFA Score Day 3	0.165
SOFA Score Day 4	0.165
SOFA Score Day 5	0.176
SOFA Score Day 6	0.322
SOFA Score Day 7	0.297

Variable in SVM7 Day Model	Mean AUC loss
SOFA Score Day 1	0.172
SOFA Score Day 2	0.191
SOFA Score Day 3	0.171
SOFA Score Day 4	0.170
SOFA Score Day 5	0.170
SOFA Score Day 6	0.187
SOFA Score Day 7	0.218

B)

Permutation-based Feature Importance(1) for aNN 5d and SVM 5d

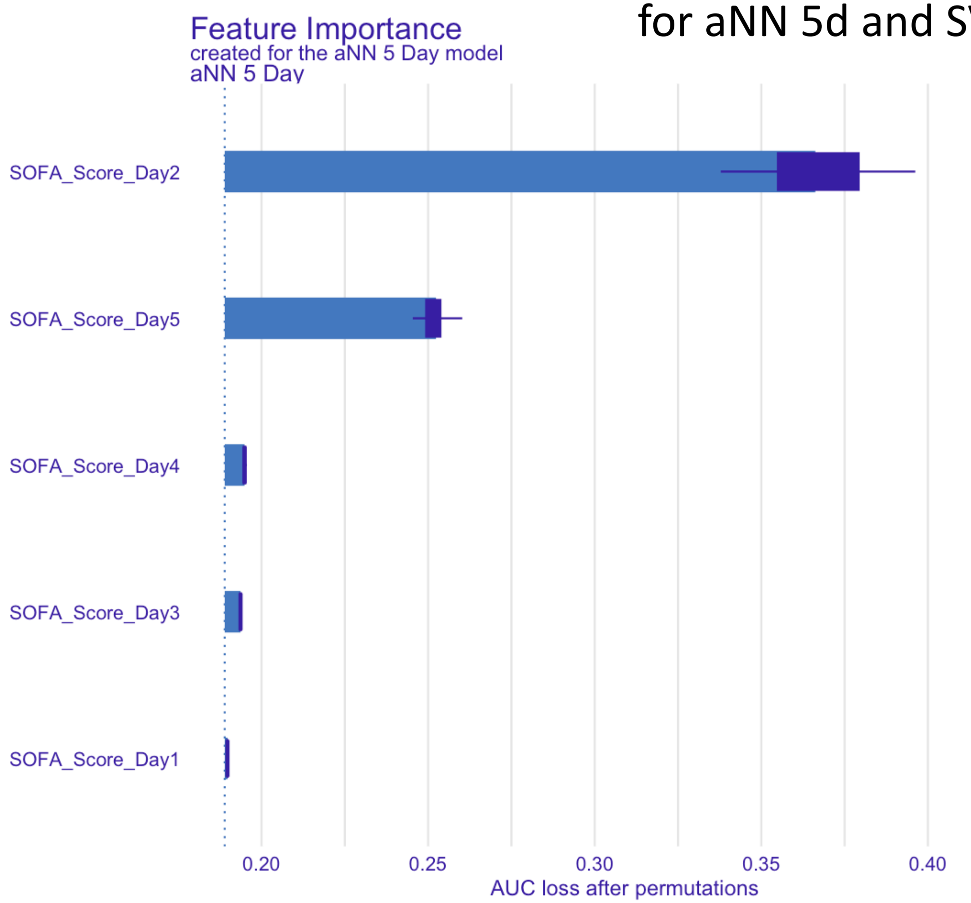

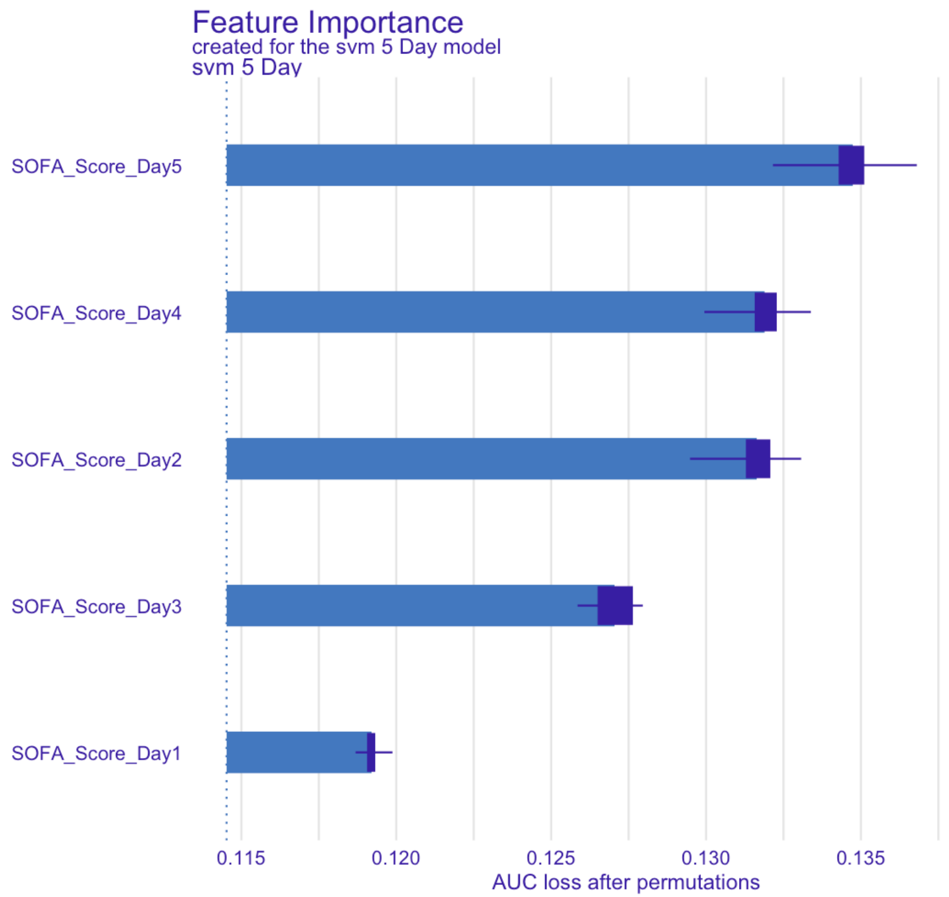

Variable in aNN5 Day Model	Mean AUC loss
SOFA Score Day 1	0.190
SOFA Score Day 2	0.366
SOFA Score Day 3	0.194
SOFA Score Day 4	0.195
SOFA Score Day 5	0.252

Variable in SVM5 Day Model	Mean AUC loss
SOFA Score Day 1	0.118
SOFA Score Day 2	0.131
SOFA Score Day 3	0.1127
SOFA Score Day 4	0.132
SOFA Score Day 5	0.135

C)

Permutation-based Feature Importance[1] for aNN 3d and SVM 3d

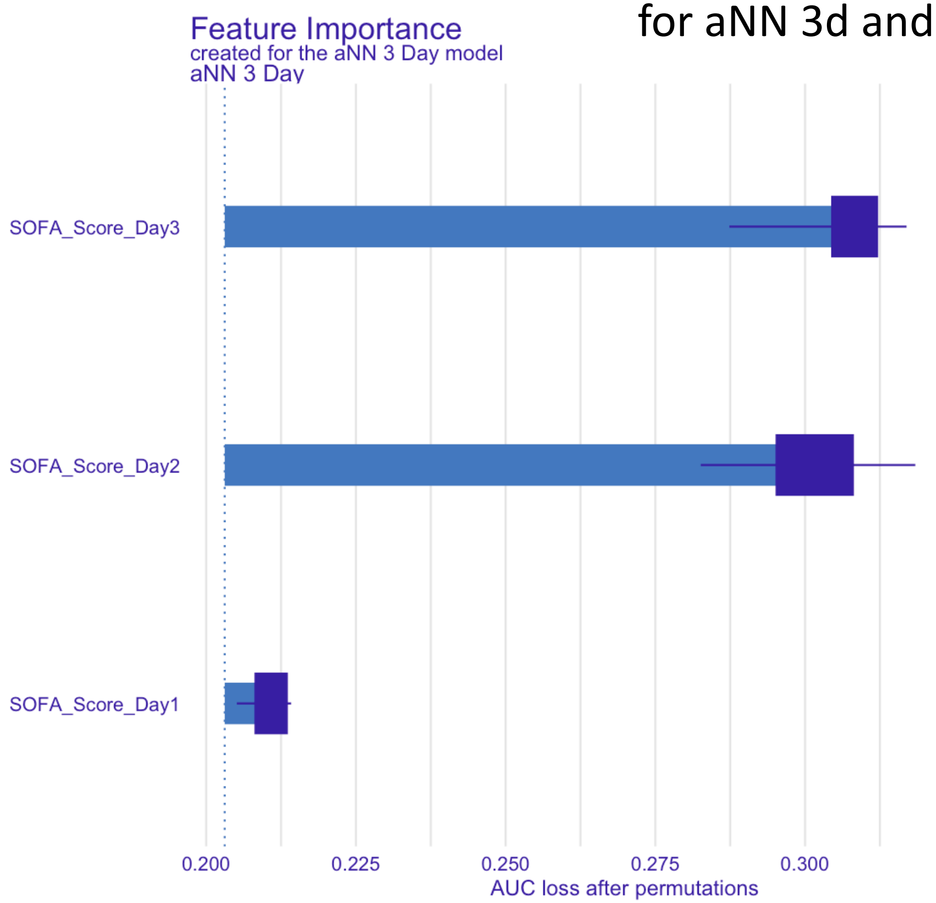

Variable in aNN7 Day Model	Mean AUC loss
SOFA Score Day 1	0.211
SOFA Score Day 2	0.301
SOFA Score Day 3	0.307

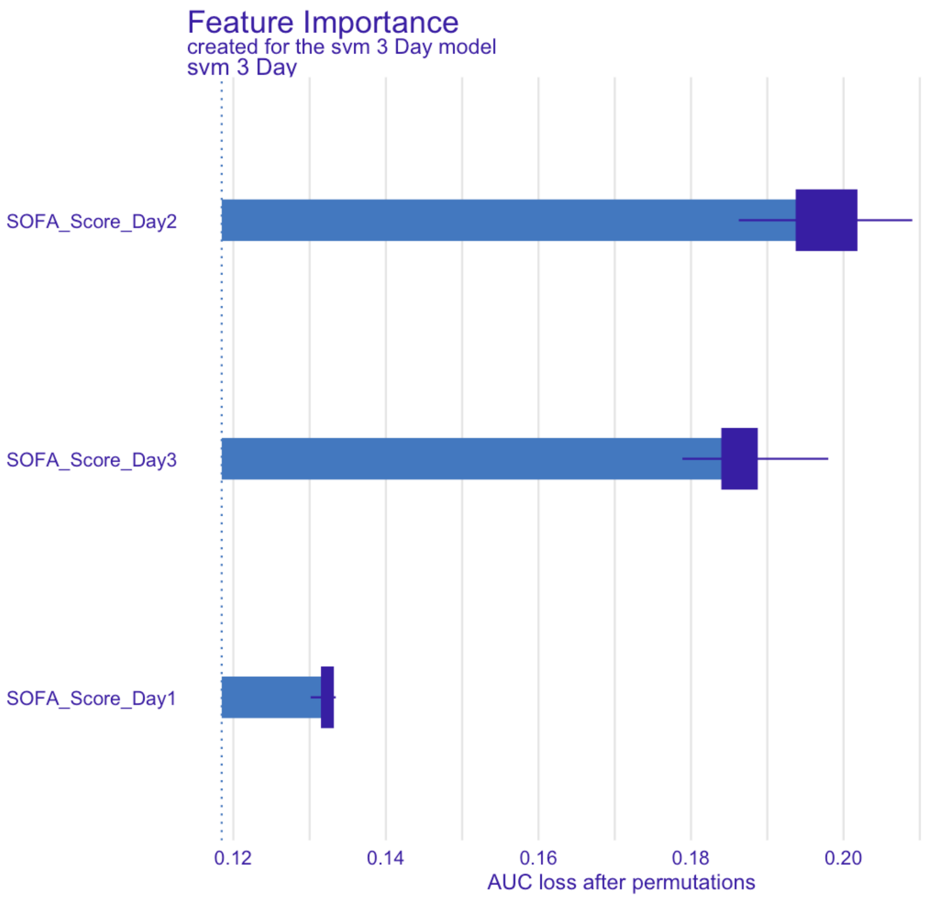

Variable in SVM7 Day Model	Mean AUC loss
SOFA Score Day 1	0.132
SOFA Score Day 2	0.201
SOFA Score Day 3	0.189

D)

Example Case #1

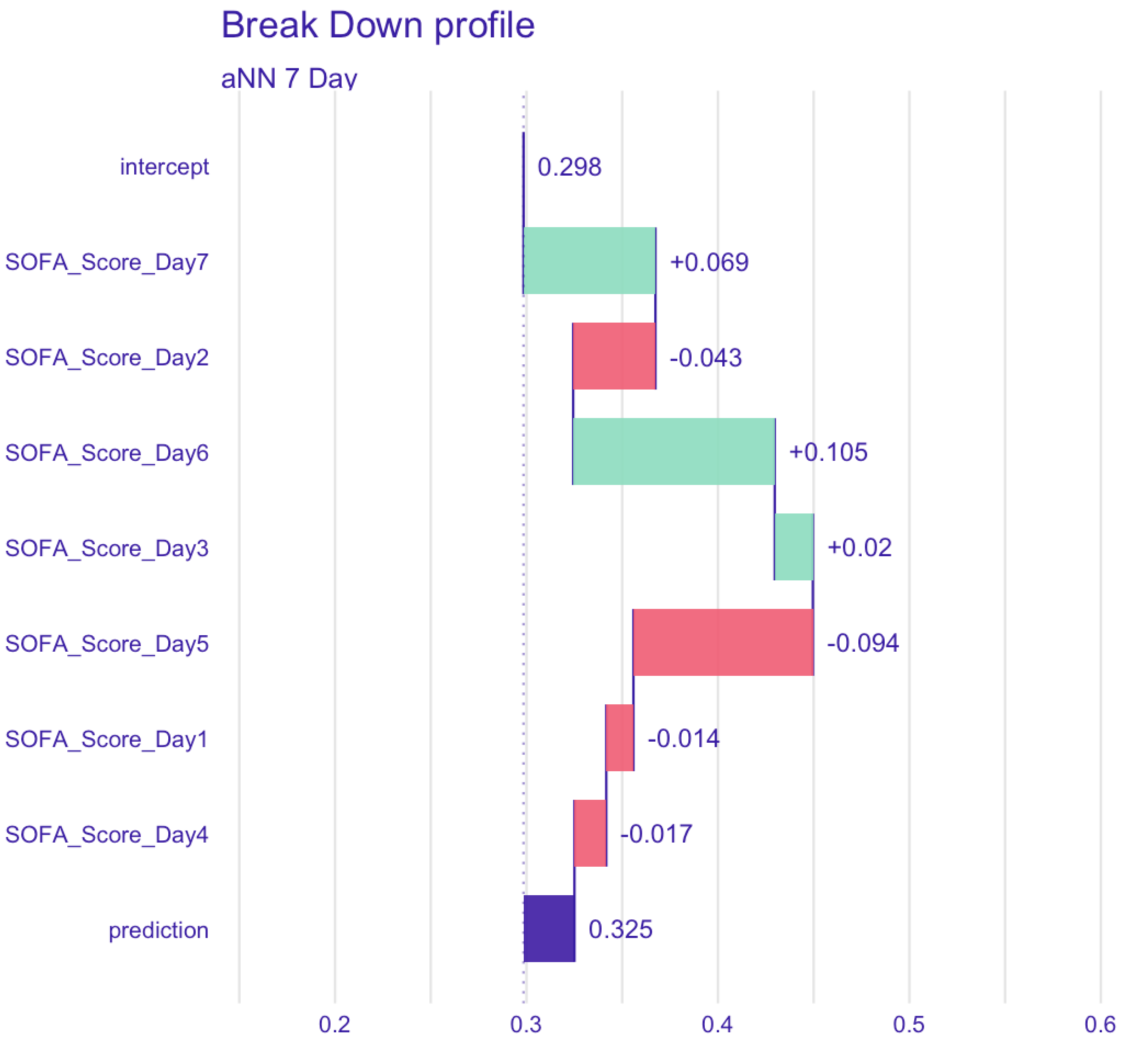

SOFA Score	Value
Day 1	7
Day 2	7
Day 3	5
Day 4	8
Day 5	9
Day 6	8
Day 7	9

aNN 7	Probability
Predicted death	0.325

Presenting SHAP(2) value for each variable in Case#15 of Derivation cohort of aNN 7d (survived)

E)

Example Case #2

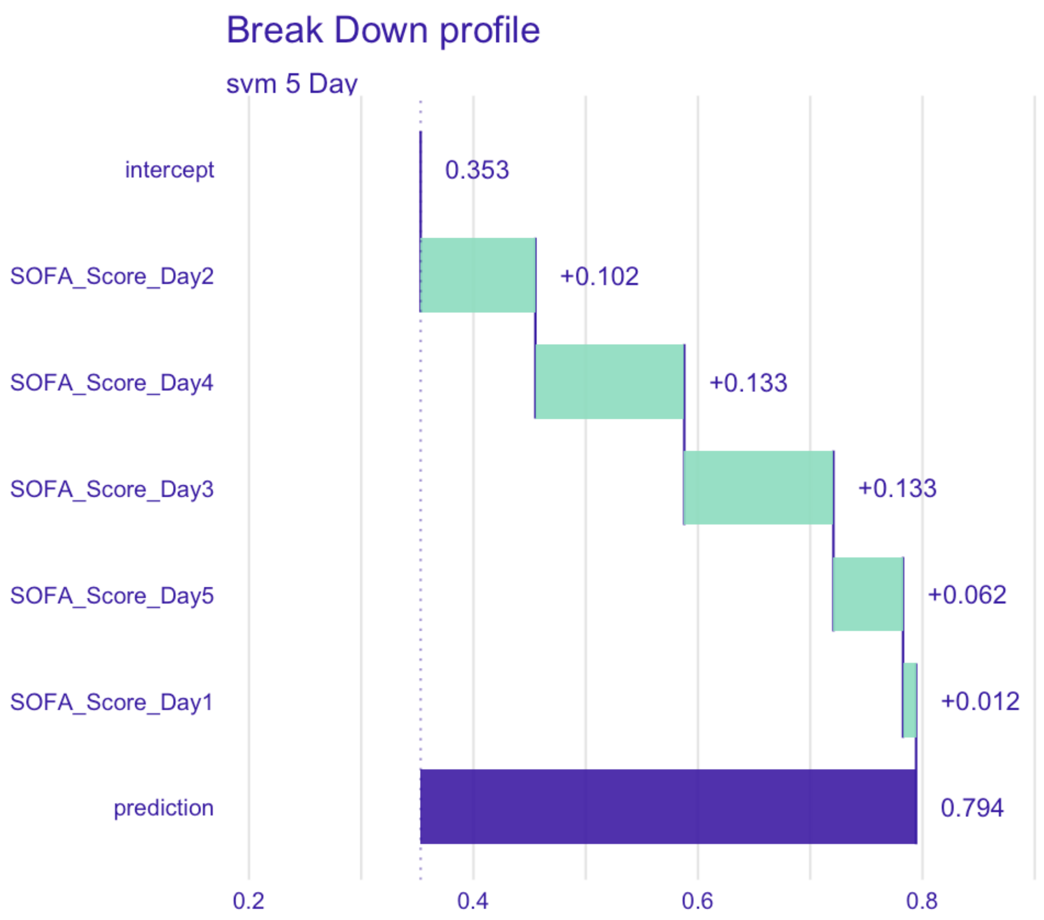

Presenting SHAP (2) value for each variable in Case#199 of Derivation cohort of SVM 5d (deceased)

SOFA Score	Value
Day 1	9
Day 2	14
Day 3	7
Day 4	13
Day 5	9

SVM 5	Probability
Predicted death	0.794

References:
1. Mi X, Zou B, Zou F, Hu J. Permutation-based identification of important biomarkers for complex diseases via machine learning models. *Nat Commun*. 2021;12(1):3008. doi:10.1038/s41467-021-22756-2

2. Rodríguez-Pérez R, Bajorath J. Interpretation of machine learning models using shapley values: application to compound potency and multi-target activity predictions. *J Comput Aided Mol Des*. 2020;34(10):1013-1026. doi:10.1007/s10822-020-00314-0
