# Supplementary material for: Assessing SOFA score trajectories in sepsis using machine learning: A pragmatic approach to improve the accuracy of mortality prediction
Source: PLoS One. 2024 Mar 28;19(3):e0300739. doi: 10.1371/journal.pone.0300739 (PMC10977876; doi:10.1371/journal.pone.0300739)
Supplement: S4 File — Kaplan Meier curves, cross tables and proportion of survived patients for the a) ΔSOFA score and b) artificial neural network (aNN). 1: Day 1–5. 2: Day 1–3. (PDF) [file pone.0300739.s004.pdf]

1) Kaplan Meier curves, cross tables and proportion of survived patients for the a) ΔSOFA score (day 1 and day 5) and b) artificial neural network (aNN) (day 1 till day 5)

A) ΔSOFA Day 1 to 5

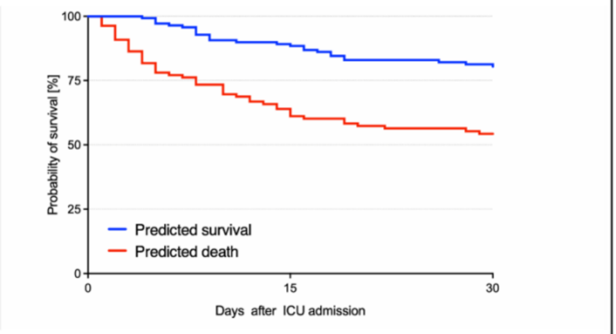

	PREDICTED SURVIVAL	PREDICTED DEATH
**SURVIVED**	116	61
**DECEASED**	26	49
**SURVIVAL PROPORTIONS AFTER 30 DAYS**	81%	54%

B) aNN Day 1 to 5

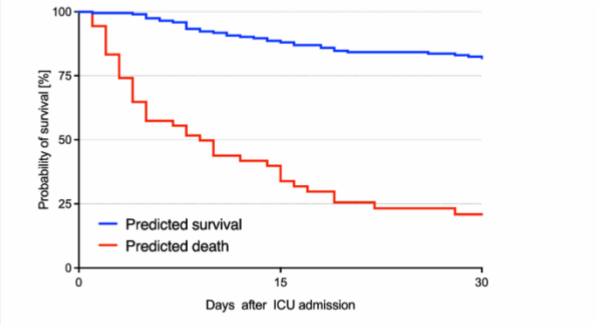

	PREDICTED SURVIVAL	PREDICTED DEATH
**SURVIVED**	164	13
**DECEASED**	34	41
**SURVIVAL PROPORTIONS AFTER 30 DAYS**	82%	21%

2) Kaplan Meier curves, cross tables and proportion of survived patients for the a) ΔSOFA score (day 1 and day 3) and b) artificial neural network (aNN) (day 1 till day 3)

A) ΔSOFA Day 1 to 3

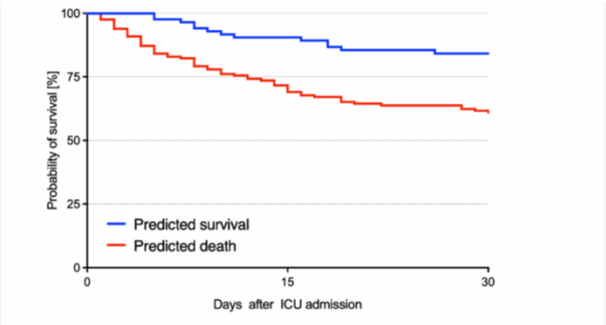

	PREDICTED SURVIVAL	PREDICTED DEATH
**SURVIVED**	74	103
**DECEASED**	13	62
**SURVIVAL PROPORTIONS AFTER 30 DAYS**	84%	61%

B) aNN Day 1 to 3

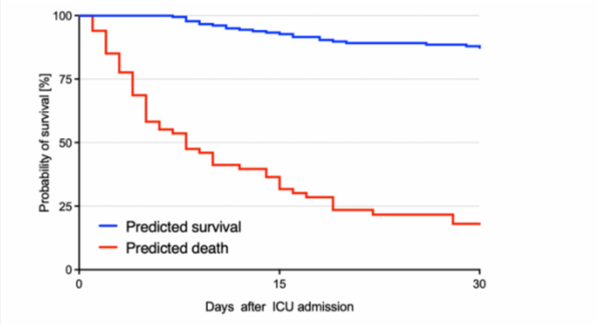

	PREDICTED SURVIVAL	PREDICTED DEATH
**SURVIVED**	166	11
**DECEASED**	32	43
**SURVIVAL PROPORTIONS AFTER 30 DAYS**	82%	19%
