# Supplementary material for: Assessing SOFA score trajectories in sepsis using machine learning: A pragmatic approach to improve the accuracy of mortality prediction
Source: PLoS One. 2024 Mar 28;19(3):e0300739. doi: 10.1371/journal.pone.0300739 (PMC10977876; doi:10.1371/journal.pone.0300739)
Supplement: S2 Table — (DOCX) [file pone.0300739.s006.docx]

Supp.-Table 1: Performance of ΔSOFA and machine learning algorithms in primary (training) cohort and validation cohort

|  |  | **Primary cohort** | | | **Validation cohort** | | |
| --- | --- | --- | --- | --- | --- | --- | --- |
|  | **Algorithm** | **AUC** | **95% CI** | **Statistically different to delta SOFA** | **AUC** | **95% CI** | **Statistically different to primary cohort** |
| **Day 1 to 3** | **ΔSOFA** | 0.661 | 0.584 – 0.738 | N/A | 0.642 | 0.612 – 0.672 | p = 0.65 |
|  | **SVM with polynomial kernel** | 0.816 | 0.684 – 0.947 | p < 0.01* | 0.776 | 0.721 – 0.832 | p = 0.25 |
|  | **SVM with linear kernel** | 0.810 | 0.676 – 0.942 | p < 0.01* | 0.779 | 0.724 – 0.835 | p = 0.38 |
|  | **Neural net** | 0.797 | 0.661 – 0.933 | p < 0.01* | 0.766 | 0.710 – 0.823 | p = 0.39 |
|  | **Neural Networks with Feature Extraction** | 0.817 | 0.686 – 0.948 | p < 0.01* | 0.771 | 0.715 – 0.827 | p = 0.19 |
|  | **Logistic Regression** | 0.816 | 0.676 – 0.942 | p < 0.01* | 0.779 | 0.723 – 0.834 | p = 0.29 |
|  | **LDA** | 0.811 | 0.678 – 0.943 | p < 0.01* | 0.779 | 0.724 – 0.835 | p = 0.36 |
|  | **Random Forest** | 0.773 | 0.631 – 0.914 | p = 0.03* | 0.670 | 0.608 – 0.733 | p < 0.01***** |
| **Day 1 to 5** | **ΔSOFA** | 0.695 | 0.620 – 0.770 | N/A | 0.673 | 0.643 – 0.703 | p = 0.59 |
|  | **SVM with polynomial Kernel** | 0.824 | 0.694 – 0.953 | p < 0.01* | 0.798 | 0.744 – 0.851 | p = 0.45 |
|  | **SVM with linear kernel** | 0.818 | 0.688 – 0.949 | p = 0.01* | 0.802 | 0.749 – 0.856 | p = 0.64 |
|  | **Neural net** | 0.801 | 0.666 – 0.937 | p = 0.04* | 0.799 | 0.745 – 0.852 | p = 0.96 |
|  | **Neural Networks with Feature Extraction** | 0.823 | 0.694 – 0.952 | p < 0.01* | 0.790 | 0.735 – 0.844 | p = 0.34 |
|  | **Logistic Regression** | 0.816 | 0.680 – 0.945 | p = 0.02* | 0.799 | 0.745 – 0.852 | p = 0.63 |
|  | **LDA** | 0.815 | 0.683 – 0.946 | p = 0.02* | 0.803 | 0.750 – 0.856 | p = 0.73 |
|  | **Random Forest** | 0.791 | 0.653 – 0.929 | p = 0.06 | 0.706 | 0.644 – 0.766 | p = 0.02***** |
| **Day 1 to 7** | **ΔSOFA** | 0.727 | 0.654 – 0.800 | N/A | 0.689 | 0.659 – 0.719 | p = 0.34 |
|  | **SVM with polynomial Kernel** | 0.837 | 0.712 – 0.963 | p = 0.02* | 0.819 | 0.768 – 0.871 | p = 0.59 |
|  | **SVM with linear kernel** | 0.829 | 0.701 – 0.956 | p = 0.03* | 0.820 | 0.768 – 0.871 | p = 0.79 |
|  | **Neural net** | 0.822 | 0.692 – 0.951 | p = 0.05* | 0.817 | 0.766 – 0.869 | p = 0.88 |
|  | **Neural Networks with Feature Extraction** | 0.837 | 0.712 – 0.962 | p = 0.02* | 0.812 | 0.761 – 0.865 | p = 0.45 |
|  | **Logistic Regression** | 0.828 | 0.698 – 0.955 | p = 0.04* | 0.816 | 0.764 – 0.868 | p = 0.72 |
|  | **LDA** | 0.830 | 0.703 – 0.957 | p = 0.03* | 0.820 | 0.768 – 0.871 | p = 0.77 |
|  | **Random Forest** | 0.797 | 0.661 – 0.933 | p = 0.16 | 0.731 | 0.672 – 0.791 | p = 0.07 |
