# Supplementary material for: Assessing SOFA score trajectories in sepsis using machine learning: A pragmatic approach to improve the accuracy of mortality prediction
Source: PLoS One. 2024 Mar 28;19(3):e0300739. doi: 10.1371/journal.pone.0300739 (PMC10977876; doi:10.1371/journal.pone.0300739)
Supplement: S3 Table — a) Aiming for a sensitivity of at least 90% while maximizing specificity b) Aiming for a specificity of at least 90% while maximizing sensitivity. (DOCX) [file pone.0300739.s007.docx]

Performance of aNN and SVM models focusing on sensitivity and specificity:

A) Aiming for a sensitivity of at least 90% while maximizing specificity

B) Aiming for a specificity of at least 90% while maximizing sensitivity

A)

|  | **Algorithm** | **Sensitivity** | **Specificity** |
| --- | --- | --- | --- |
| **Day 1 to 3** | **ΔSOFA** | 91% | 20% |
|  | **SVM with polynomial kernel** | 91% | 50% |
|  | **Neural network** | 91% | 43% |
| **Day 1 to 5** | **ΔSOFA** | 91% | 25% |
|  | **SVM with polynomial kernel** | 91% | 56% |
|  | **Neural network** | 91% | 44% |
| **Day 1 to 7** | **ΔSOFA** | 91% | 20% |
|  | **SVM with polynomial kernel** | 91% | 59% |
|  | **Neural network** | 91% | 58% |

B)

|  | **Algorithm** | **Sensitivity** | **Specificity** |
| --- | --- | --- | --- |
| **Day 1 to 3** | **ΔSOFA** | 25% | 91% |
|  | **SVM with polynomial kernel** | 55% | 90% |
|  | **Neural network** | 60% | 93% |
| **Day 1 to 5** | **ΔSOFA** | 37% | 90% |
|  | **SVM with polynomial kernel** | 55% | 91% |
|  | **Neural network** | 55% | 91% |
| **Day 1 to 7** | **ΔSOFA** | 29% | 90% |
|  | **SVM with polynomial kernel** | 61% | 91% |
|  | **Neural network** | 63% | 90% |
