# Supplementary material for: Assessing SOFA score trajectories in sepsis using machine learning: A pragmatic approach to improve the accuracy of mortality prediction
Source: PLoS One. 2024 Mar 28;19(3):e0300739. doi: 10.1371/journal.pone.0300739 (PMC10977876; doi:10.1371/journal.pone.0300739)
Supplement: S4 Table — (DOCX) [file pone.0300739.s008.docx]

**Breakdown of mortality within the first seven days and at the end of 30-day follow-up**

| Day | Alive, n | Deceased, n | Mortality rate, % |
| --- | --- | --- | --- |
| 1 | 248 | 4 | 1.59 % |
| 2 | 242 | 10 | 3.97 % |
| 3 | 236 | 16 | 6.35 % |
| 4 | 229 | 23 | 9.13 % |
| 5 | 221 | 31 | 12.30 % |
| 6 | 218 | 34 | 13.49 % |
| 7 | 215 | 37 | 14.68 % |
| 30 | 177 | 76 | 30.16 % |
