# Supplementary material for: Assessing SOFA score trajectories in sepsis using machine learning: A pragmatic approach to improve the accuracy of mortality prediction
Source: PLoS One. 2024 Mar 28;19(3):e0300739. doi: 10.1371/journal.pone.0300739 (PMC10977876; doi:10.1371/journal.pone.0300739)
Supplement: S5 Table — (DOCX) [file pone.0300739.s009.docx]

Performance of ΔSOFA and machine learning algorithms in validation cohort stratified by admission intervals

|  |  | **2003 to 2007**  (n = 458) | | **2008 to 2012**  (n = 621) | | **2013 to 2016**  (n = 711) | |
| --- | --- | --- | --- | --- | --- | --- | --- |
|  | **Algorithm** | **AUC** | **95% CI** | **AUC** | **95% CI** | **AUC** | **95% CI** |
| **Day 1 to 3** | **ΔSOFA** | 0,652 | 0,609 - 0,696 | 0,651 | 0,602 - 0,702 | 0,626 | 0,567 - 0,688 |
|  | **SVM with polynomial kernel** | 0,785 | 0,715 - 0,859 | 0,786 | 0,708 - 0,868 | 0,763 | 0,687 - 0,842 |
|  | **SVM with linear kernel** | 0,778 | 0,709 - 0,851 | 0,788 | 0,711 - 0,870 | 0,784 | 0,706 - 0,865 |
|  | **Neural net** | 0,772 | 0,701 - 0,846 | 0,781 | 0,702 - 0,865 | 0,764 | 0,687 - 0,845 |
|  | **Neural Networks with Feature Extraction** | 0,773 | 0,703 - 0,846 | 0,786 | 0,707 - 0,869 | 0,768 | 0,691 - 0,848 |
|  | **Logistic Regression** | 0,788 | 0,716 - 0,860 | 0,768 | 0,691 - 0,847 | 0,788 | 0,709 - 0,868 |
|  | **LDA** | 0,766 | 0,697 - 0,837 | 0,786 | 0,709 - 0,868 | 0,788 | 0,710 - 0,871 |
|  | **Random Forest** | 0,661 | 0,588 - 0,738 | 0,679 | 0,597 - 0,765 | 0,671 | 0,591 - 0,756 |
| **Day 1 to 5** | **ΔSOFA** | 0,685 | 0,643 - 0,728 | 0,662 | 0,613 - 0,712 | 0,662 | 0,614 - 0,713 |
|  | **SVM with polynomial Kernel** | 0,797 | 0,728 - 0,867 | 0,786 | 0,711 - 0,863 | 0,813 | 0,735 - 0,893 |
|  | **SVM with linear kernel** | 0,809 | 0,741 - 0,881 | 0,805 | 0,729 - 0,885 | 0,794 | 0,719 - 0,873 |
|  | **Neural net** | 0,794 | 0,726 - 0,864 | 0,802 | 0,726 - 0,880 | 0,791 | 0,715 - 0,869 |
|  | **Neural Networks with Feature Extraction** | 0,790 | 0,721 - 0,860 | 0,788 | 0,711 - 0,867 | 0,786 | 0,709 - 0,865 |
|  | **Logistic Regression** | 0,808 | 0,738 - 0,879 | 0,812 | 0,734 - 0,892 | 0,785 | 0,710 - 0,862 |
|  | **LDA** | 0,804 | 0,736 - 0,874 | 0,796 | 0,719 - 0,876 | 0,809 | 0,733 - 0,889 |
|  | **Random Forest** | 0,715 | 0,639 - 0,791 | 0,695 | 0,615 - 0,776 | 0,698 | 0,617 - 0,780 |
| **Day 1 to 7** | **ΔSOFA** | 0,692 | 0,648 - 0,736 | 0,685 | 0,635 - 0,736 | 0,682 | 0,633 - 0,733 |
|  | **SVM with polynomial Kernel** | 0,815 | 0,749 - 0,884 | 0,803 | 0,731 - 0,879 | 0,817 | 0,744 - 0,894 |
|  | **SVM with linear kernel** | 0,804 | 0,738 - 0,871 | 0,815 | 0,740 - 0,892 | 0,831 | 0,755 - 0,909 |
|  | **Neural net** | 0,812 | 0,746 - 0,881 | 0,824 | 0,751 - 0,901 | 0,801 | 0,729 - 0,878 |
|  | **Neural Networks with Feature Extraction** | 0,810 | 0,744 - 0,880 | 0,828 | 0,753 - 0,909 | 0,806 | 0,732 - 0,884 |
|  | **Logistic Regression** | 0,818 | 0,751 - 0,888 | 0,812 | 0,737 - 0,890 | 0,831 | 0,754 - 0,910 |
|  | **LDA** | 0,829 | 0,761 - 0,898 | 0,828 | 0,752 - 0,906 | 0,829 | 0,753 - 0,907 |
|  | **Random Forest** | 0,721 | 0,650 - 0,796 | 0,740 | 0,662 - 0,823 | 0,719 | 0,641 - 0,801 |
